# Drug delivery of 6-bromoindirubin-3’-glycerol-oxime ether employing poly(d,l-lactide-co-glycolide)-based nanoencapsulation techniques with sustainable solvents

**DOI:** 10.1186/s12951-021-01179-7

**Published:** 2022-01-04

**Authors:** Anna Czapka, Christian Grune, Patrick Schädel, Vivien Bachmann, Karl Scheuer, Michael Dirauf, Christine Weber, Alexios-Leandros Skaltsounis, Klaus D. Jandt, Ulrich S. Schubert, Dagmar Fischer, Oliver Werz

**Affiliations:** 1 Department of Pharmaceutical/Medicinal Chemistry, Institute of Pharmacy, Friedrich Schiller University Jena, Philosophenweg 14, 07743 Jena, Germany; 2 Pharmaceutical Technology and Biopharmacy, Institute of Pharmacy, Friedrich Schiller University Jena, Lessingstraße 8, 07743 Jena, Germany; 3 Chair of Materials Science (CMS), Faculty of Physics and Astronomy, Otto Schott Institute of Materials Research, Friedrich Schiller University Jena, Löbdergraben 32, 07743 Jena, Germany; 4 Laboratory of Organic and Macromolecular Chemistry (IOMC), Friedrich Schiller University Jena, Humboldtstraße 10, 07743 Jena, Germany; 5 Department of Pharmacy, Division of Pharmacognosy and Natural Products Chemistry, University of Athens, Panepistimiopolis Zografou, 15771 Athens, Greece; 6 Jena Center for Soft Matter (JCSM), Friedrich Schiller University Jena, Philosophenweg 7, 07743 Jena, Germany; 7 Division of Pharmaceutical Technology, Department for Chemistry and Pharmacy, Friedrich-Alexander-University Erlangen-Nürnberg, Cauerstrasse 4, 91058 Erlangen, Germany

**Keywords:** Nanoparticles, Drug delivery, PLGA, Sustainable solvents, Inflammation, Indirubin

## Abstract

**Background:**

Insufficient solubility and stability of bioactive small molecules as well as poor biocompatibility may cause low bioavailability and are common obstacles in drug development. One example of such problematic molecules is 6-bromoindirubin-3'-glycerol-oxime ether (6BIGOE), a hydrophobic indirubin derivative. 6BIGOE potently modulates the release of inflammatory cytokines and lipid mediators from isolated human monocytes through inhibition of glycogen synthase kinase-3 in a favorable fashion. However, 6BIGOE suffers from poor solubility and short half-lives in biological aqueous environment and exerts cytotoxic effects in various mammalian cells. In order to overcome the poor water solubility, instability and cytotoxicity of 6BIGOE, we applied encapsulation into poly(d,l-lactide-co-glycolide) (PLGA)-based nanoparticles by employing formulation methods using the sustainable solvents Cyrene™ or 400 g/mol poly(ethylene glycol) as suitable technology for efficient drug delivery of 6BIGOE.

**Results:**

For all preparation techniques the physicochemical characterization of 6BIGOE-loaded nanoparticles revealed comparable crystallinity, sizes of about 230 nm with low polydispersity, negative zeta potentials around − 15 to − 25 mV, and biphasic release profiles over up to 24 h. Nanoparticles with improved cellular uptake and the ability to mask cytotoxic effects of 6BIGOE were obtained as shown in human monocytes over 48 h as well as in a shell-less hen’s egg model. Intriguingly, encapsulation into these nanoparticles fully retains the anti-inflammatory properties of 6BIGOE, that is, favorable modulation of the release of inflammation-relevant cytokines and lipid mediators from human monocytes.

**Conclusions:**

Our formulation method of PLGA-based nanoparticles by applying sustainable, non-toxic solvents is a feasible nanotechnology that circumvents the poor bioavailability and biocompatibility of the cargo 6BIGOE. This technology yields favorable drug delivery systems for efficient interference with inflammatory processes, with improved pharmacotherapeutic potential.

**Graphical Abstract:**

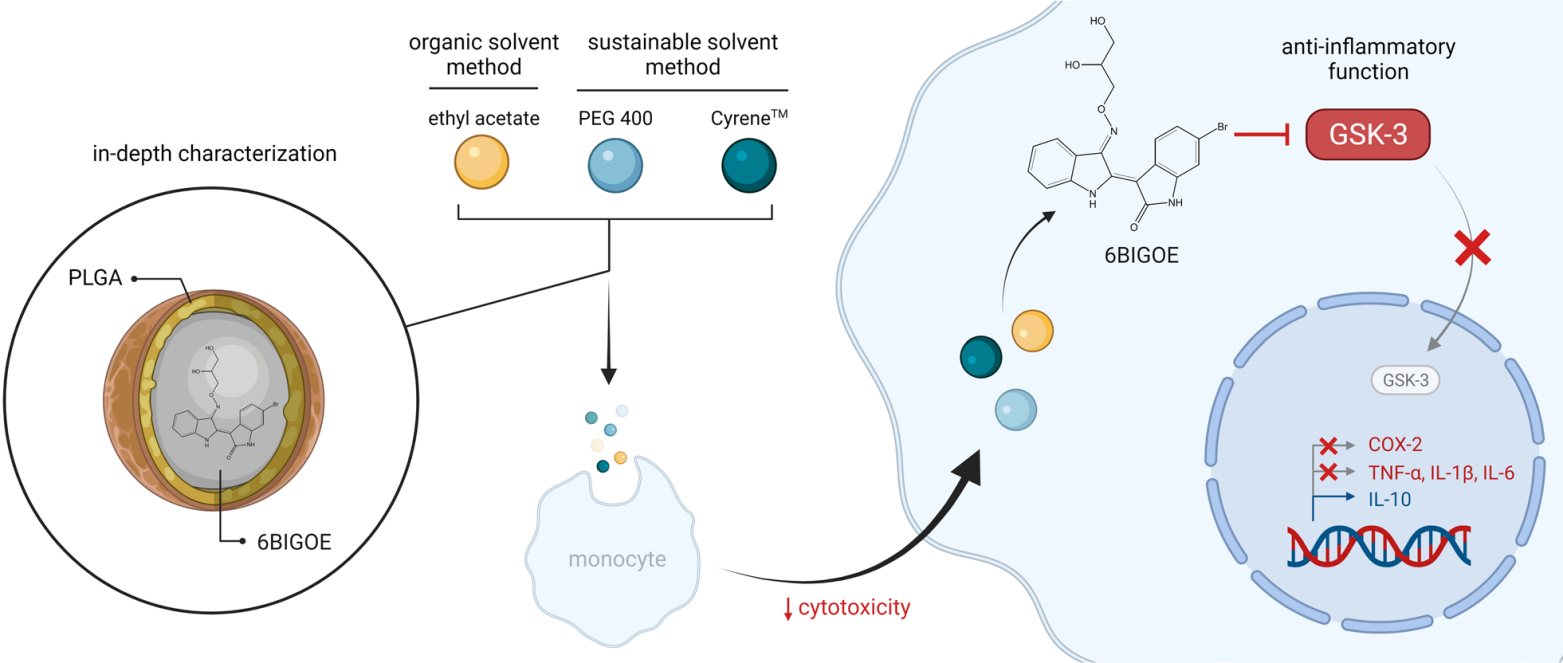

**Supplementary Information:**

The online version contains supplementary material available at 10.1186/s12951-021-01179-7.

## Background

Natural products have a longstanding tradition as pharmacological agents, for example in Traditional Chinese Medicine. Indirubin is a bioactive ingredient contained in the formulation *Danggui Longhui Wan* which is used for the treatment of chronic myeloid leukemia [1]. Indirubin and its synthetic analogues are well-established inhibitors of cyclin-dependent kinases, dual-specificity tyrosine-regulated kinases, aurora kinases, and glycogen synthase kinase (GSK)-3 [2–4], and therefore offer a broad therapeutic potential for the treatment of various disorders ranging from inflammatory [5, 6], autoimmune [7], metabolic [8] and neurodegenerative diseases [9] to cancer [10]. Among these indirubins, 6-bromoindirubin-3'-glycerol-oxime ether (6BIGOE) was identified as a highly potent and favorable modulator of inflammatory cytokines and lipid mediators (LMs) in human monocytes and thus, represents an interesting candidate for the treatment of inflammation-related diseases [11]. However, 6BIGOE and its close structural derivative 6-bromoindirubin-3'-oxime (6BIO) exhibit cytotoxic effects [3, 12, 13] and suffer from poor water solubility, low stability, and short half-life times in blood [11, 14, 15].

Application of bioactive small molecules is often impeded by their poor aqueous solubility [16] and the requirement of potentially harmful organic solvents which demands the use of suitable drug delivery systems [17]. Thus, administration of such bioactive drugs in vivo is limited [18] and their pharmaceutical potential is often overlooked. A variety of techniques is available to enhance solubility, including crystal engineering, use of surfactants, complexation [16], or application of nanocarrier systems [19]. Recently, optimized delivery by a mixed micellar formulation was shown for the transdermal passage of an indirubin analog [20], and a self-nanoemulsifying drug delivery system successfully increased oral bioavailability of an indirubin derivative by 984% in vivo [18]. Thus, application of nanoparticle (NP) drug delivery systems is a suitable biotechnology to increase bioavailability of hydrophobic drugs. Additionally, drug encapsulation can avoid unintended off-target interactions and distribution in vivo*,* and increase circulation time [21].

For applications in humans, commercially available poly(lactic-*co*-glycolic acid) (PLGA) are commonly used as biodegradable polymers that are approved in many applications by the Food and Drug Administration and European Medicine Agency [22, 23]. Due to their excellent pharmaceutical features, such as protection of cargo from degradation and achievement of high biocompatibility, while offering controlled drug release and tailored biodegradability, PLGA is a highly suitable candidate for the formulation of nanoparticulate carrier systems [23]. Standard techniques for the preparation of NPs are emulsion-diffusion-evaporation (EDE) and nanoprecipitation [24]. In most cases, the use of organic solvents, such as ethyl acetate and other class II or III category solvents (categorized by the International Council for Harmonisation of Technical Requirements for Pharmaceuticals for Human Use [25]) is required [26]. This leads to environmental risks, problematic handling and health risks for the operator, in particular in upscaled industrial settings [27]. Therefore, there is a huge demand for alternative, non-toxic replacements that meet additional criteria such as miscibility with water, non-volatility, low viscosity as well as good drug and solvent stability [28]. Recent studies focus on alternative approaches applying non-toxic, sustainable solvents such as PEG 400 [29]. PLGA-NPs prepared with this method were successfully used to encapsulate the lipophilic atorvastatin and improved the anti-inflammatory capacity of the drug [29]. Recently, a commercially available solvent raised particular interest, that is, Cyrene™ (dihydrolevoglucosenone) [30]. Cyrene is a cellulose-based solvent that is easily synthesized in an almost carbon neutral process and is highly biodegradable. We successfully used Cyrene as solvent for the preparation of PLGA-NPs encapsulating atorvastatin, proving as fast and convenient, non-toxic formulation method with no plasticizing effects [30].

Intrigued by previous results, we aimed at investigating if encapsulation of the lipophilic, anti-inflammatory 6BIGOE into PLGA-NPs is feasible and offers beneficial effects with respect to bioavailability, cytotoxicity and pharmacological profile. Differences and similarities of encapsulation approaches using the organic solvent ethyl acetate and sustainable solvents such as PEG 400 and Cyrene were revealed by in-depth characterization of physicochemical parameters. We assessed the uptake of NPs into human primary monocytes, cytotoxic effects and biocompatibility, as well as biological functionality. Our data show that PLGA-NPs, independent of the preparation method, are useful as drug delivery system for 6BIGOE, which retains its potent ability to interfere with the inflammation process by modulating pro- and anti-inflammatory mediators.

## Results

### Nanoparticle preparation and characterization

Three different preparation techniques were used for the formulation of 6BIGOE with a molar mass of 430.13 g/mol and a calculated log P value of 2.24 into PLGA-NPs [31]. A standard EDE method using ethyl acetate substituted with PEG 400 was applied as reference technique in comparison to two alternative methods using the sustainable solvents PEG 400 [29, 32] and the cellulose-derived Cyrene [30]. The alternative methods were already successfully applied for the fabrication of PLGA-NPs loaded with proteins such as lysozyme and bovine serum albumin [32], and the small lipophilic molecule atorvastatin [29, 30], favoring not only the advantage of sustainability, but also a faster and easier process handling [29].

The formulation techniques differ not only in their solvents, but also in the homogenization techniques. Whereas both, the EDE and Cyrene method used high energy homogenization techniques like Ultra-Turrax and ultrasonication, respectively, the PEG 400 method worked only with magnetic stirring. However, due to the high viscosity of PEG 400, preparation at 37 °C was necessary to obtain suitable NP properties, which however, can lead to stability problems of thermosensitive drugs [29].

**Fig. 1:**
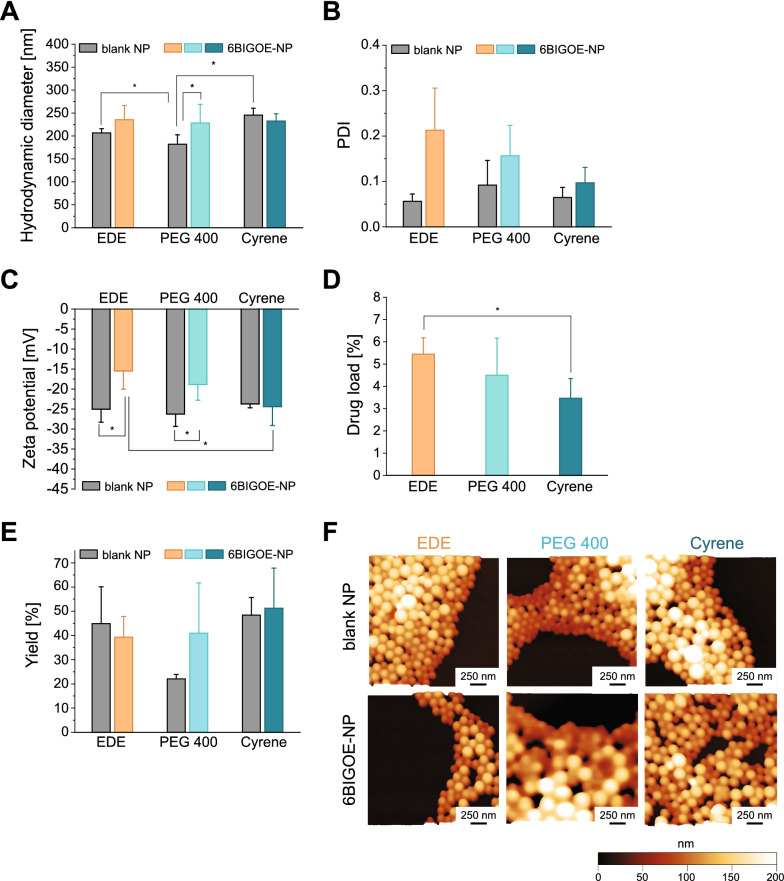
Characterization of 6BIGOE-loaded NPs. Bar charts comparing the particle size (hydrodynamic diameter, **A**), polydispersity index (PDI, **B**), zeta potential (ZP, **C**), drug load (DL, **D**), yield (**E**) and morphology (**F**) of blank or 6BIGOE-loaded NPs, prepared by standard emulsion-diffusion-evaporation (EDE), Cyrene, or PEG 400 method. Samples were analyzed by DLS techniques in water (HD, PDI, ZP) or after lyophilization (yield, DL). DL was determined by UV/Vis spectroscopy at 520 nm. Results are reported as means ± SD in three independent batches. (**F**) Atomic force microscopy height images of blank NP and 6BIGOE-loaded NP prepared by standard EDE, Cyrene, or PEG 400 method. NP dispersion was drop casted on freshly cleaned mica and allowed to dry before analysis

The particle size was determined by photon correlation spectroscopy showing hydrodynamic diameters (HD) between 182 and 245 nm (Fig. 1A). Blank NP sizes increased in the order PEG 400 < EDE < Cyrene with larger particles for the Cyrene method. Encapsulation of 6BIGOE adapted the particle sizes for all preparation methods comparably to about 230 nm. All samples showed a polydispersity index (PDI) below 0.21, indicating a narrow particle size distribution. The PDI slightly increased through the encapsulation of 6BIGOE from 0.06–0.09 for blank NPs to 0.10–0.21 for 6BIGOE-loaded NPs, but still was below 0.21. The increase was only significant for the EDE NPs (Fig. 1B), which was observed for paclitaxel-loaded PLGA NPs before [33].

The zeta potential (ZP) of the NPs can prevent nanoparticle aggregation due to electrostatic repulsion [34]. Blank particles of all formulations showed comparable ZP between − 23.7 mV and − 27.3 mV similar to former studies [29, 30, 35]. An increase of the ZP was obtained for 6BIGOE-loaded NPs prepared by the EDE and PEG 400 method with values of − 15.5 mV and − 18.9 mV, respectively, whereas 6BIGOE-loaded NPs prepared by the Cyrene method showed a ZP similar to blank NPs (Fig. 1C). The values of the ZP correlated with the drug load (DL) as determined by UV/Vis spectrophotometric measurements. The higher the DL (3.5 to 5.5%), the less negative was the ZP which might be related to the essentially neutral character of 6BIGOE at pH 7 (Fig. 1D).

The 6BIGOE-loaded NPs showed a yield in the range of 39 to 51% without significant differences between the preparation methods and to the blank NPs, except blank PEG 400 NPs (22%) (Fig. 1E). The morphology of the NPs was visualized by atomic force microscopy (AFM) imaging and height images showed spherical, smooth and homogenous particles without open pores. Analysis of particle height indicated a monomodal size distribution of the NPs with mean values of 106.8 ± 21.3 nm and 121.3 ± 24.5 nm (EDE), 86.3 ± 37.2 nm and 153.6 ± 39.2 nm (PEG 400), 128.8 ± 33.3 nm and 117.9 ± 20.1 nm (Cyrene) for blank NPs and 6BIGOE-loaded NPs, respectively (Additional file 1: Fig. S2). Note that the height determined by AFM is lower than the HD determined by dynamic light scattering (DLS), which can be attributed to the NPs collapsing during the drying process [36]. These findings are supported by the results obtained from the laser light scattering measurements showing a monomodal size distribution (Additional file 1: Fig. S3). In case of the NPs obtained from PEG 400, a small film on the particles can be noticed, which might be PEG 400 residues from NP formulation (Fig. 1F).

### Nanoparticle thermal behavior and in vitro 6BIGOE release

**Fig. 2:**
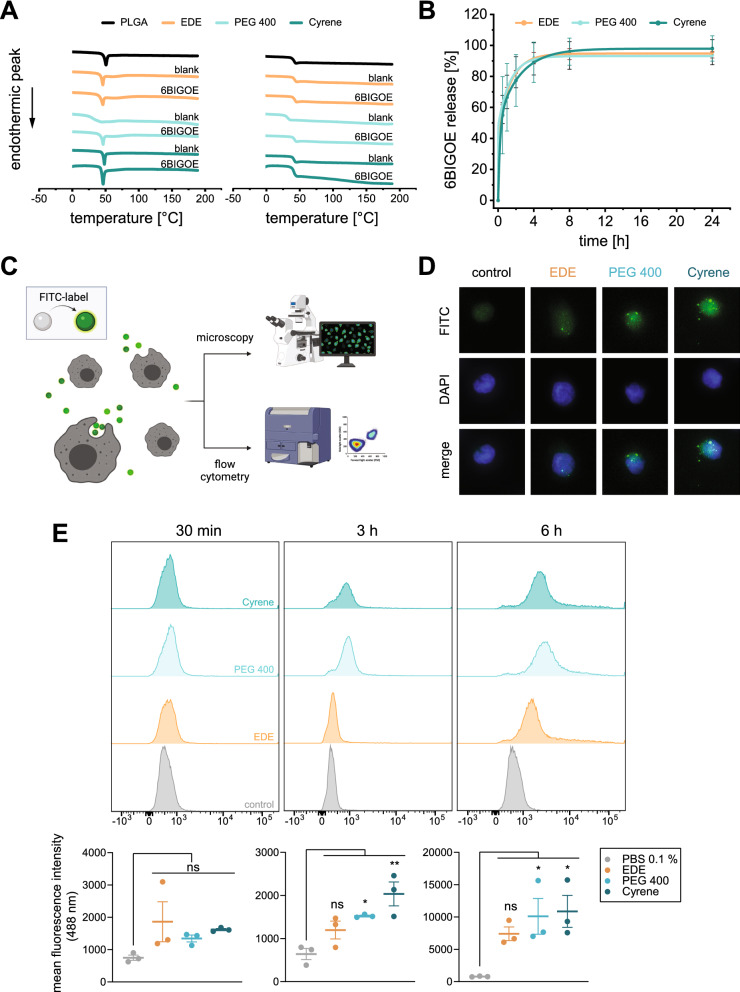
NP behavior: drug release and NP uptake. **A** Thermal behavior of blank NPs and 6BIGOE-loaded NPs prepared by emulsion-diffusion-evaporation (EDE), Cyrene, or PEG 400 method analyzed by DSC. Two heating–cooling cycles at 10 °C/min were applied for run 1 (left) and run 2 (right), shown for the range between 0 to 190 °C. **B** Cumulative in vitro drug release from 6BIGOE-loaded NPs prepared by EDE, Cyrene, or PEG 400 method in PBS / Tween 80 (1% (m/m)) pH 7.4, up to 168 h. 6BIGOE was quantified by UV/Vis spectroscopy at 520 nm, data are means ± SD of three independent NP batches. **C** Simplified workflow depicting analysis of fluorescence-labeled NP uptake by human monocytes. **D**,** E** Uptake of fluorescein-labeled 6BIGOE-loaded NPs in human monocytes was analyzed using microscopy and flow cytometry. Monocytes (0.8 × 10^6^/mL (**D**), 1 × 10^6^/mL (**E**)) were incubated for the indicated times with 6BIGOE-loaded NPs prepared by EDE, Cyrene, or PEG 400 method. **D** Fluorescence microscopy of monocytes visualizing uptake of fluorescein-labeled 6BIGOE-loaded NPs by monocytes after 3 h. Individual fluorescence channel for FITC (upper panel) and overlay with DAPI nuclear stain (lower panel) are shown. Results presented for one single cell are representative for approx. 100 individual cells analyzed in three independent experiments. **E** Representative histograms (upper panel) display uptake of fluorescein-labeled 6BIGOE-loaded NPs by CD14^+^ monocytes (PBS 0.1%, negative control). Data are presented as mean fluorescent intensity for 30 min, 3 h or 6 h (lower panel); n = 3 separate donors, one-way ANOVA for multiple comparisons with Dunnett’s correction

To investigate the NP thermal behavior depending on the type of solvent in the O-phase, which could influence biological aspects like drug release, cell uptake, and solubility of the drug [37–39], differential scanning calorimetry (DSC) analysis was performed. The free polymer and most of the NPs showed a sharp peak during the first heating run. Due to the polymer being amorphous and the glass transition temperature (T_g_) determined in the second run being very similar, we concluded that these are not endothermic melting peaks. Presumably the sharp peaks are a result of polymeric chains of the polymer and the NPs undergoing a relaxation process upon reaching T_g_. Therefore, a detailed evaluation of the T_g_ was made from the second run (Fig. 2A and Additional file 1: Table S2). The free PLGA polymer per se showed a T_g_ of 41.4 °C which is in accordance with the supplier (Sigma-Aldrich) information (i.e., 42 to 46 °C, for Resomer^®^ RG 502, poly(d,l-lactide-co-glycolide). Comparable T_g_ of 42.7 and 41.2 °C were obtained for blank NPs prepared by the EDE and Cyrene method, respectively, whereas for those prepared with the PEG 400 method a T_g_ of 33.9 °C and no sharp peak could be observed pointing to an amorphous modification of the NPs. This is in accordance with a recent study, where blank and atorvastatin-containing NPs prepared with RG 502 and 505 showed a similar reduction of the T_g_ due to the plasticizing effect of PEG [29]. The incorporation of 6BIGOE resulted in T_g_s of about 41.1 to 42.3 °C for all preparations, independent of the type of applied solvent. This is comparable to the blank NPs with exception of the PEG 400 method where the presence of 6BIGOE counteracted the plasticizing effects of PEG 400 resulting in a higher T_g_. The in vitro drug release of 6BIGOE was investigated for up to 168 h using phosphate-buffered saline (PBS) plus 1% Tween 80 (m/m) (pH 7.4) as release medium which showed a sufficient solubility for 6BIGOE (29.34 µg/ml) and accomplished sink conditions in the present setting. Under the chosen conditions and dependent on the selected buffer system, a fast and almost complete drug release could be observed for NPs of all formulations techniques already after 4 h (Fig. 2B).

### Uptake of fluorescence-labeled 6BIGOE-NPs prepared by different formulation methods in human monocytes

To visualize the PLGA-NPs in cellular uptake studies, fluorescein-labelled PLGA-NPs with or without 6BIGOE were prepared using all methods under identical conditions. In order to introduce the label, commercial Resomer^®^ RG502H was modified with *N*-hydroxy succinimide and subsequently coupled to an amino-functionalized fluorescein-5-isothiocyanate (FITC) derivative (Additional file 1: SI1 and Fig. S1). Acid terminated Resomer^®^ RG502H was used due to its carboxylic group that was necessary for coupling. To remove residual non-conjugated FITC, the reaction mixture was purified by precipitation in diethyl ether and subsequently methanol, followed by dialysis against water. The successful covalent attachment of the label was proven by means of size-exclusion chromatography (SEC), simultaneously detecting the refractive index (RI) and Vis signal (wavelength = 463 nm, Additional file 1: Fig. S1) of the polymer. The obtained signals were overlapping, which can only be the case if the dye was successfully attached to the polymer. The non-conjugated FITC derivative was additionally characterized by SEC for referencing of its elution volume (Additional file 1: Fig. S1). Its signal slightly overlapped with the RI signal of the non-functionalized polymer, which is probably due to the broad dispersity of the PLGA. However, the presence of significant amounts of free dye in the purified FITC-PLGA can be excluded as the Vis signals of the free label and the labelled PLGA are almost baseline separated in the elugram. For NP preparation, a mixture of FITC-PLGA and PLGA (Resomer^®^ RG 502, 1:4) was used. FITC-PLGA-NPs were characterized, results are summarized in Additional file 1: Table S1. In comparison to PLGA-NPs without FITC labeling, the FITC-PLGA-NP showed comparable particle properties.

To investigate the influence of the preparation technique on the uptake of NPs, we incubated human primary monocytes with the fluorescently-labeled NPs and monitored their temporal uptake employing either fluorescence microscopy or flow cytometry (Fig. 2C). After incubation of monocytes with 6BIGOE-loaded NPs for 3 h, their uptake was first visualized by fluorescence microscopy. Although the cellular uptake was observed for all types of NPs, the NPs prepared using the PEG 400 or the Cyrene method seemed to enrich in larger amounts compared with NP formulated by the EDE method (Fig. 2D).

Next, we incubated the monocytes with fluorescently labeled 6BIGOE-loaded NPs and monitored the cellular uptake by flow cytometry after 30 min, 3 h and 6 h. All histograms revealed shifts in NP uptake over time (Fig. 2E). After 30 min, there was little to no uptake observable from the histogram, which is in agreement with the mean fluorescent intensity revealing no significant changes comparing 6BIGOE-loaded NPs to the control or between formulation methods. After 3 h, the uptake of NPs prepared by EDE method was not markedly enhanced compared to 30 min incubation. Interestingly, 6BIGOE-loaded NPs prepared by methods using sustainable solvents, such as the PEG 400 or Cyrene, displayed a significant increase in uptake compared to NPs generated by the EDE method. After 6 h, NP uptake was strongly enhanced when NPs were prepared by the PEG 400 or Cyrene method. Surprisingly, the uptake of NPs prepared by EDE was not significantly enhanced at 6 h versus 3 h, though a tendency for some increased enrichment was obvious. Together, NPs prepared by methods using sustainable solvents are favorably taken up by human primary monocytes.

### Encapsulation of 6BIGOE into NPs prevents detrimental effects on cell viability and governs biocompatibility

It is known that 6BIGOE and related indirubin derivatives display cytotoxic effects against cancer cells and non-transformed human cells [3, 12, 13]. Although selective cytotoxicity of a given compound is a desired feature for cancer therapy, detrimental effects on cell viability are generally not favorable for the treatment of inflammatory disorders. Encapsulation of 6BIGOE into NPs may prevent unwanted interactions of the drug with extracellular off-targets and premature degradation. However, import of particles in the nm-scale into cells or tissues, may cause irritating effects and demands for careful evaluation of biocompatibility.

**Fig. 3:**
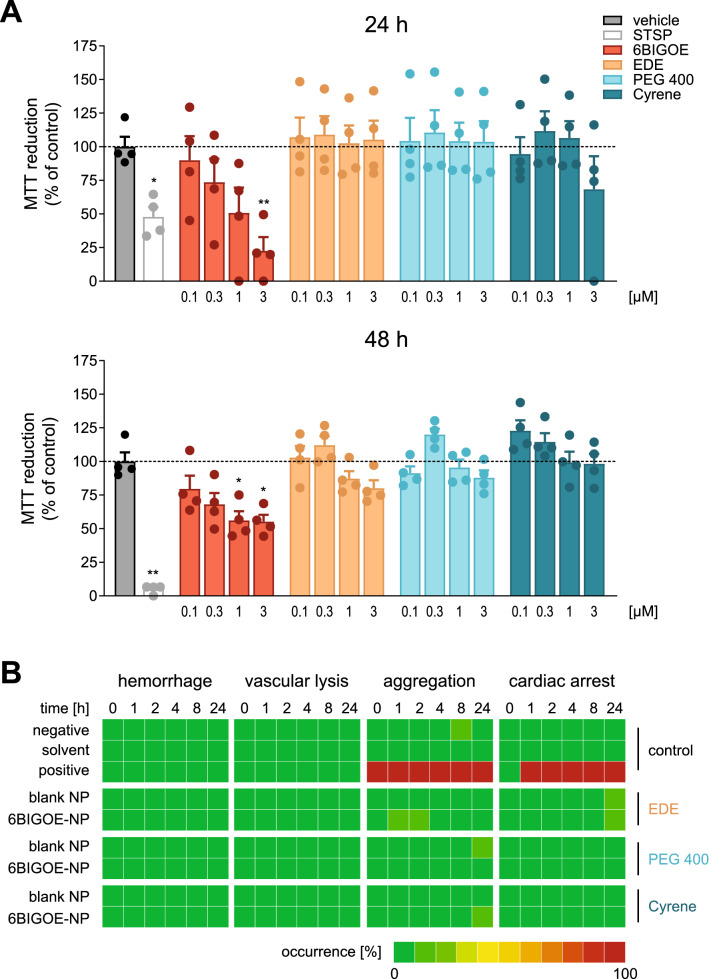
Assessment of cytotoxic potential and biocompatibility of blank and 6BIGOE-loaded NPs. **A** Cell viability of human monocytes was evaluated by MTT assay. Cells were treated for 24 or 48 h with vehicle (DMSO 0.5% (v/v) for 6BIGOE or PBS 0.1% (v/v) for 6BIGOE-loaded NPs), 6BIGOE, 6BIGOE-loaded NPs prepared by emulsion-diffusion-evaporation (EDE), Cyrene, or PEG 400 method at the indicated 6BIGOE concentrations, or 1 µM staurosporine (STSP, positive control), at 37 °C, and MTT assay was performed. Values are means + SEM; expressed as percentage of control (vehicle = 100%); n = 4 separate donors. Statistical analysis was performed applying repeated-measurement one-way ANOVA with Geisser-Greenhouse correction and Dunnett’s multiple comparisons test, testing treatments against vehicle (0.1% DMSO for 6BIGOE and STSP or 0.1% PBS for NPs). Data was log-transformed prior to analysis for 6BIGOE-loaded NPs at 24 h. (**B**) Biocompatibility assessment of the blank and 6BIGOE-loaded NPs in an *ex ovo* shell-less hens egg test. The clustergram illustrates toxic effects of injected (2 µL) 6BIGOE-loaded NPs prepared by EDE, Cyrene, or PEG 400 method. A concentration of 3 µM 6BIGOE calculated on the 6BIGOE drug load was used. Deionized water was used as solvent control, 0.9% NaCl served as negative control and branched PEI was applied as positive control. The columns quantify the time-dependent effect, whereas the rows show the different test samples. The number of affected egg’s correlates to the intensity of the color. Data were collected in two independent experiments with a total number of 10 eggs per NP sample

To assess detrimental effects of 6BIGOE and 6BIGOE-loaded NPs prepared by different formulation methods, we analyzed the viability of human monocytes after 24 or 48 h incubations using the 3-(4,5-dimethylthiazol-2-yl)-2,5-diphenyltetrazolium bromide (MTT) assay. While after 24 h the apoptosis-inducer staurosporine (STSP, 1 µM) significantly decreased cell viability, no significant cytotoxic effects were evident for 6BIGOE at concentrations up to 1 µM, although 6BIGOE significantly reduced cell viability at 3 µM in agreement with results obtained before [11]. Notably, 6BIGOE-NPs prepared by either formulation method did not affect monocyte viability within 24 h over the complete concentration range up to 3 µM (Fig. 3A). After 48 h of treatment, STSP caused strong cytotoxicity as expected, and also 6BIGOE decreased cell viability with comparable concentration–response pattern and even higher potency as observed at 24 h, being significantly cytotoxic at 1 µM (Fig. 3A). Interestingly, these marked cytotoxic properties of 6BIGOE were attenuated by encapsulation of the drug into NPs regardless of the preparation method (Fig. 3A). Blank NPs displayed no cytotoxic effects when compared with PBS (0.1%) control for 24 h and 48 h (Additional file 1: Fig. S4).

In vitro cytotoxicity is typically investigated in cell culture models limited by the absence of a dynamic blood flow and the complex interaction between blood cells, plasma proteins, and the endothelium. Therefore, the in vivo situation can often not be properly assessed by simple 2D cell culture models [40]. The shell-less hen’s egg model on the chick area vasculosa (CAV) offers a more complex biological system with a dynamic blood flow closer to the in vivo situation and favors the advantage of a fast performance and cost-efficiency [41]. Samples were applied by microinjection into the vitelline vein of the eggs. We previously demonstrated the biocompatibility of 6BIGOE at 3 µM in the hen’s egg model [11]. Therefore, 3 µM 6BIGOE was selected in the present study for NP testing, calculated on the DL of 6BIGOE. To exclude unspecific NP effects, blank NPs were injected at the same concentrations. The eggs were investigated for different toxic effects like hemorrhage (bleeding into the surrounding tissue), vascular lysis (decay of the blood vessels), aggregation of blood components, and cardiac arrest.

As shown in Fig. 3B and in accordance with the historical lab values, the negative control (0.9% NaCl) and the solvent control (deionized water) did not provoke any toxic effects (0–1/10 eggs). Branched poly(ethylene imine) (PEI, 25 g/mol, 25 mg/mL in deionized water) as positive control showed a strong aggregation of blood components already during injection which resulted in the cardiac arrest of all eggs after one hour. NPs prepared by all three methods showed no toxicity with only minor aggregation of blood components (blank NPs (Cyrene) and 6BIGOE-loaded NPs (EDE and PEG 400)) and cardiac arrest (blank and 6BIGOE-loaded NPs (EDE)) in < 10% of the eggs. Thus, all NPs can be considered as biocompatible independent of the preparation method and the DL.

### 6BIGOE in NPs retains the capacity to potently modulate cytokine release from human monocytes

**Fig. 4.**
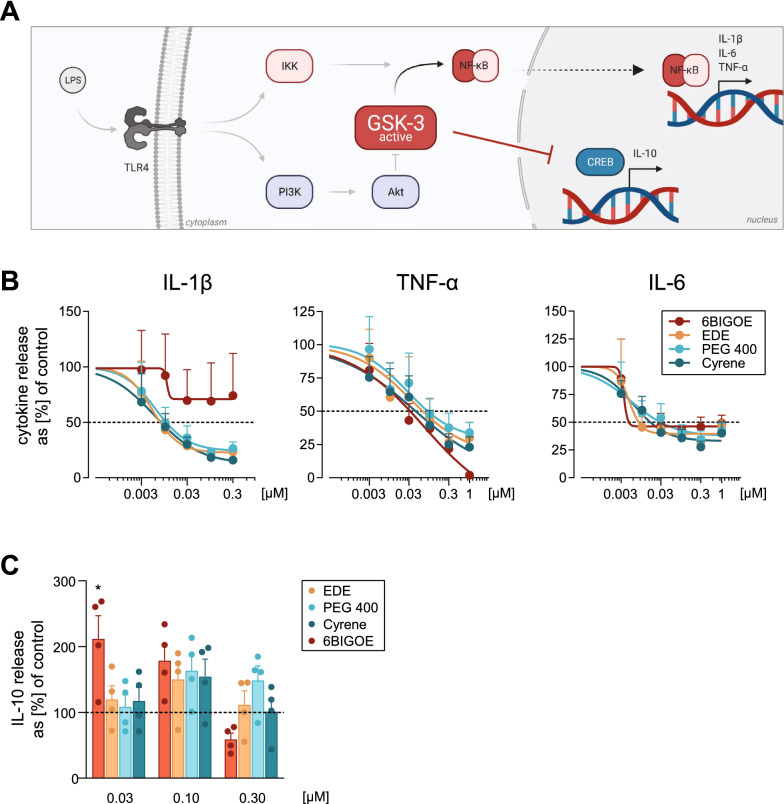
6BIGOE and 6BIGOE-loaded NPs modulate LPS-induced cytokine release from monocytes. **A** LPS-induced TLR4 stimulation results in modulation of pro- and anti-inflammatory cytokine formation via GSK-3 activity and associated downstream signaling pathways in human monocytes. **B**,** C** Human monocytes (10^6^/mL) were pretreated for 3 h with vehicle (0.1% DMSO for 6BIGOE or equivalent amount of blank NP dispersions), 6BIGOE or 6BIGOE-loaded NPs prepared by EDE, Cyrene, or PEG 400 method in a concentration-dependent manner (0.003 to 1 µM) as indicated, and subsequently stimulated with 100 ng/ml LPS for 18 h. (**B**) Release of TNF-α, IL-β, and IL-6 was determined by ELISA. Values are means + SEM; expressed as percentage of control (vehicle = 100%), n = 4 separate experiments. Statistical analysis was conducted on log-transformed data using one-way ANOVA with Holm-Šídák correction for multiple comparisons of control group (DMSO 0.1%) with 6BIGOE treatments. Unpaired, two-sided Student’s *t*-test was applied for 6BIGOE-loaded NP treatments and their respective NP blank controls. **C** Release of IL-10 was determined by ELISA. Values are means + SEM; expressed as percentage of control (vehicle = 100%). n = 4 separate donors. Statistical analysis was conducted applying one-way ANOVA with Dunnett’s correction for multiple comparisons of 6BIGOE at indicated concentrations with control (DMSO 0.1%), and unpaired, two-sided Student’s *t*-test for comparison of different 6BIGOE-NP treatments with their respective controls (NP blanks)

Apart from being non-cytotoxic and able to transport the cargo into target cells, delivery systems also need to accomplish the effective release of the cargo upon arrival at the intracellular destination, where the compound is expected to exert its biological function. Thus, we assessed the anti-inflammatory effects of free 6BIGOE and 6BIGOE-loaded NP in monocytes that are known to be caused by inhibition of GSK-3 [11]. Upon stimulation with lipopolysaccharide (LPS), the Toll-like receptor 4 (TLR4) is activated and initiates immune responses via downstream routes, especially via GSK-3 and nuclear factor (NF)-κB pathways (Fig. 4A). 6BIGOE as GSK-3 inhibitor most likely interferes with inflammatory signaling pathways by increasing nuclear CREB DNA-binding activity and by simultaneously blocking NF-κB subunit p65 binding to DNA, resulting in decreased levels of the pro-inflammatory cytokines interleukin (IL)-1β, tumor necrosis factor (TNF)-α and IL-6, and in promotion of the release of anti-inflammatory IL-10 [11, 42]. We employed an established experimental setup [11] to analyze the modulation of these inflammation-related cytokines by 6BIGOE and 6BIGOE-loaded NPs in human monocytes upon 3 h pre-incubation and subsequent LPS challenge. As expected [11], pretreatment with 6BIGOE effectively reduced the levels of all pro-inflammatory cytokines, starting at very low concentrations of about 0.003 µM to 0.01 µM (Fig. 4B). Comparable concentration-dependent effects were observed for 6BIGOE-loaded NPs, independent of the encapsulation method used (Fig. 4B). Of interest, the suppression of IL-1β release was more potent for 6BIGOE-loaded NPs compared with free 6BIGOE. Note that in this treatment regimen free 6BIGOE caused only partial suppression of IL-1β release, as compared to our previous study [11]. Similarly, release of TNF-α and IL-6, was concentration-dependently (0.003 to 1 µM) suppressed by all types of 6BIGOE-loaded NPs with comparable efficiencies as for free 6BIGOE (Fig. 4B). Compared to TNF-α levels, reduction of IL-6 started at slightly higher concentrations with incomplete suppression for free or encapsulated 6BIGOE. In agreement with our previous results [11], 6BIGOE (0.03 and 0.1 µM) stimulated the release of anti-inflammatory IL-10 (Fig. 4C). Encapsulated 6BIGOE at 0.1 µM showed the same ability, regardless of the NP formulation method used. Taken together, encapsulation of 6BIGOE into PLGA-NPs as delivery systems by applying different formulation methods accomplishes efficient and beneficial modulation of inflammation-related cytokines in human monocytes without loss of potency versus the free drug.

### 6BIGOE-loaded NPs modulate lipid mediator formation via inhibition of COX-2 expression

**Fig. 5.**
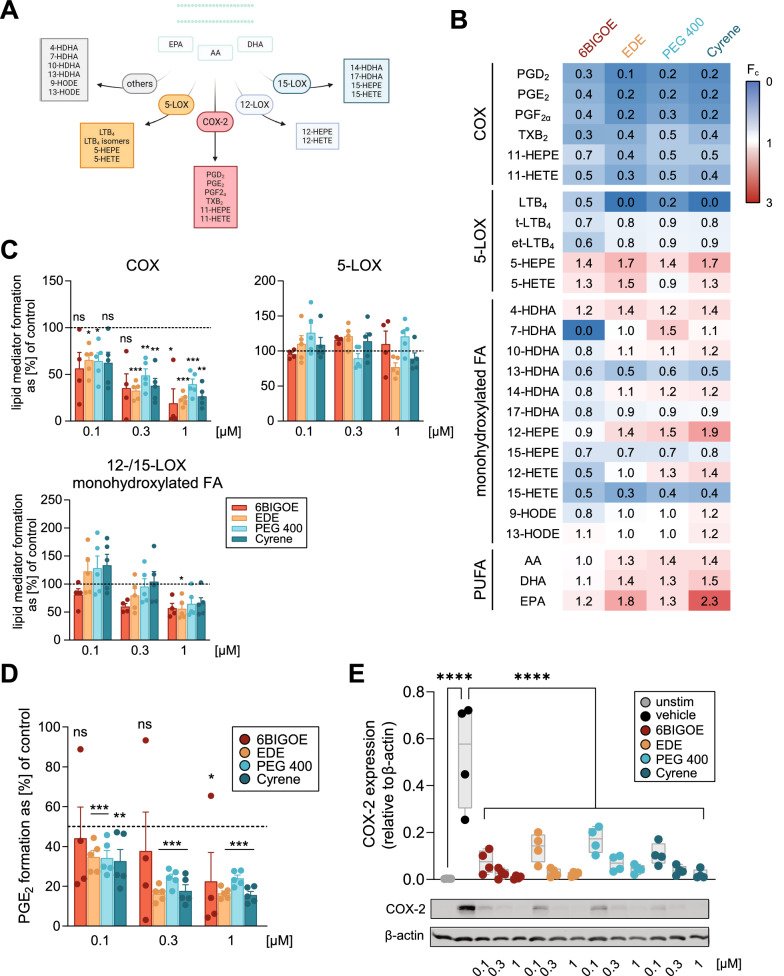
6BIGOE-loaded NPs downregulate prostanoid biosynthesis by inhibition of COX-2 expression in monocytes. **A** Schematic overview of LM biosynthetic pathways. **B**-**D** Human monocytes (10^6^/mL) were pretreated with vehicle (0.1% DMSO for 6BIGOE, or equivalent amount of blank NP dispersions), 6BIGOE or 6BIGOE-loaded NPs prepared by EDE, Cyrene, or PEG 400 method for 3 h before stimulation with 100 ng/mL LPS for 18 h. Formed LM were extracted from the supernatant by SPE and analyzed by UPLC-MS–MS. **B** Relative changes in the formation of single LMs from monocytes treated with 6BIGOE or 6BIGOE-loaded NPs at 0.3 µM versus vehicle-treated cells are shown in a heatmap, n = 5 separate donors, except 6BIGOE n = 4. Corresponding values ± SEM are included in Additional file 1: Figs. S5-S7. **C** Relative changes of LM groups in monocytes treated with 6BIGOE or 6BIGOE-loaded NPs versus vehicle-treated cells, n = 5 separate donors, except 6BIGOE n = 4. Statistical analysis was performed to compare 6BIGOE-treated cells with vehicle-treated cells (DMSO 0.1%) by one-way ANOVA with Dunnett’s correction on log-transformed data, and unpaired, two-sided Student’s *t*-test to compare 6BIGOE-loaded NPs with respective blank NP controls. **D** PGE_2_ formation in 6BIGOE- or 6BIGOE-loaded NPs-treated monocytes vs. vehicle-treated monocytes; n = 5 separate donors, except 6BIGOE n = 4. Statistical analysis was performed on log-transformed data using one-way ANOVA with Dunnett’s correction for multiple comparisons of 6BIGOE with control (DMSO 0.1%). Unpaired, two-tailed Student’s *t*-test was applied for comparison of 6BIGOE-NPs with respective NP blank controls. **E** COX-2 protein expression was assessed by Western Blot and densitometric analysis thereof, normalized to β-actin. Results are individual values represented in a box and whisker plot, n = 4 separate donors. Statistical analysis displayed was performed using one-way ANOVA for multiple comparisons with Holm-Šídák post-hoc test

Besides cytokines, de novo biosynthesized LMs are crucial signaling molecules involved in the onset, maintenance, and resolution of the inflammatory response [43]. Upon cell activation, membrane phospholipids are cleaved by phospholipases to liberate free arachidonic acid, eicosapentaenoic acid, and docosahexaenoic acid as substrates for conversion by cyclooxygenase (COX) and lipoxygenase (LOX) pathways to different LMs (Fig. 5A). Since 6BIGOE suppressed COX-derived LM formation in LPS-challenged monocytes [11], we investigated how encapsulation of 6BIGOE into PLGA-NPs affects LM biosynthesis in these cells. Individual LM profiles are displayed in a heatmap indicating the fold-change by the drug compared to vehicle control (Fig. 5B). LM and their monohydroxylated precursors are grouped into products formed by COX, 5-LOX, 12-/15-LOX and PUFAs, whereas other monohydroxylated PUFAs generated by CYPs, autoxidation or linolenic acid-derived compounds are considered as “others”. For initial screening, 6BIGOE was used at 0.3 µM as free drug or encapsulated in PLGA-NPs prepared by EDE, PEG 400, or Cyrene method. LM metabololipidomic profiling using ultra-performance liquid chromatography-tandem mass spectrometry (UPLC-MS–MS) was employed to determine signature LM footprints [44]. Overall, similar trends after pretreatment of LPS-activated monocytes with free 6BIGOE and 6BIGOE-loaded NPs were evident for the LM profiles with marked modulation of COX-derived products (Fig. 5B, Additional file 1: Figs. S5-S7). Thus, all COX products were strongly downregulated, while other LM were less or not at all affected. The potent suppressive effect of 6BIGOE on COX product formation was not hampered but rather enhanced by 6BIGOE encapsulation into PLGA-NPs, except for TXB_2_, independent of the preparation method (Fig. 5B). Interestingly and in agreement with previous findings [5], 6BIGOE, especially when encapsulated in NPs, inhibited formation of the 5-LOX-derived LTB_4_. 6BIGOE-loaded NPs increased the formation of some monohydroxylated PUFAs, such as 7-HDHA, 10-HDHA, 14-HDHA, 12-HEPE, 12-HETE, where 6BIGOE induced a decrease or no changes.

Modulation of LM formation by free or encapsulated 6BIGOE was then assessed at varying concentrations for respective LM groups: COX products include: PGD_2_, PGE_2_, PGF_2a_, TXB_2_, 11-HEPE and 11-HETE; 5-LOX products: LTB4 and LTB_4_ isomers, 5-HEPE and 5-HETE; 12-/15-LOX: monohydroxylated PUFAs such as 14-HDHA, 17-HDHA, 12-HEPE, 12-HETE, 15-HEPE and 15-HETE. Again, COX product formation was significantly decreased by 6BIGOE, especially at 0.3 and 1 µM, being concentration-dependent and overall comparable for free compound and 6BIGOE-loaded NPs with no apparent differences between preparation methods (Fig. 5C). At 0.1 µM, only 6BIGOE-NPs prepared by EDE or PEG 400 method caused significant suppression of COX products by ~ 40%. The total amount of 5-LOX products was not significantly affected, neither by 6BIGOE nor by 6BIGOE-loaded NPs, although LTB_4_ was strongly reduced by the latter. Free 6BIGOE at 0.1 and 0.3 µM moderately decreased 12/15-LOX products, in contrast to 6BIGOE-NPs that caused no alterations at these concentrations, while at 1 µM free and encapsulated 6BIGOE caused inhibitory effects (Fig. 5C).

We next focused on PGE_2_ as the most abundant and most inflammation-relevant PG and as specific marker for COX-2 activity [45]. As observed for the sum of COX products, free or encapsulated 6BIGOE concentration-dependently inhibited PGE_2_ formation in LPS-activated monocytes, with maximum inhibition of approx. 80% at 1 µM (Fig. 5D). Of note, all 6BIGOE-loaded NPs independent of the preparation method decreased PGE_2_ formation significantly at 0.1 µM, while free 6BIGOE appeared to be slightly less potent. To determine if the reduction in PGE_2_ levels was associated with inhibition of COX-2 expression by 6BIGOE as described before [11], we evaluated COX-2 protein levels in LPS-activated monocytes by Western blot. Here, strong inhibition of COX-2 expression by free and encapsulated 6BIGOE was apparent already at 0.1 µM with complete suppression at 0.3 to 1 µM (Fig. 5E). Empty NP in equivalent amounts were inactive in this respect (Additional file 1: Fig. S8). These results on COX-2 expression are in agreement with the efficient inhibition of COX product formation by 6BIGOE and PLGA-based formulations loaded with the drug.

## Discussion

Low bioavailability due to inadequate solubility and stability in an aqueous environment as well as poor biocompatibility are unifying obstacles in application of bioactive small molecules as drugs [14, 16]. One example is 6BIGOE, a highly potent anti-inflammatory derivative of the natural product indirubin which potently modulates inflammation-related cytokine and PG release from monocytes through inhibition of GSK-3 [11]. However, like other lipophilic bromo-indirubins, 6BIGOE is poorly water soluble and instable with short half-lives [15], and exhibits cytotoxic effects against a variety of cells [3, 12, 13]. Here we show that encapsulation into PLGA-NPs by employing formulation methods using sustainable solvents is a valid and suitable nanotechnology for efficient drug delivery of 6BIGOE for potential intervention with inflammatory disorders. Notably, encapsulation into NPs fully retains the anti-inflammatory properties of 6BIGOE and even represses the cytotoxic features of the drug against human monocytes as relevant target cells. Moreover, due to the apparent delay of intracellular availability of 6BIGOE when applied in NP, the favorable modulatory effects on inflammation-related cytokines and LMs may be prolonged and thus, improve the potential for anti-inflammatory pharmacotherapy.

The PLGA-NPs prepared by different formulation methods for 6BIGOE encapsulation were extensively characterized by side-by-side comparison of their physicochemical parameters, cellular uptake, biocompatibility and impact on biological activity of the cargo. We show that use of sustainable and non-toxic solvents in comparison to conventionally used organic solvents like ethyl acetate, yields comparable NPs. Intriguingly, these sustainable formulation approaches are per se faster and yield NPs with improved cellular uptake and the ability to mask cytotoxic effects of 6BIGOE despite potent interference with inflammatory processes. The HD, PDI and ZP were essentially similar for all preparation methods and comparable to previously reported results for encapsulation of atorvastatin using PEG 400 [29] or Cyrene [30]. The DL of NPs varied slightly between encapsulation methods with PEG 400 and EDE being somewhat superior over Cyrene. Such NPs with high DL could be advantageous for the application in an in vivo context, where *i.v.* application is preferable and more readily feasible with less concentrated NP dispersions. Most of the drug might be encapsulated within the PLGA matrix through strong hydrophobic interactions (Additional file 1: Fig. S9), which was previously shown for the distribution and interaction of lipophilic drugs like prednisolone in combination with PLGA by molecular dynamics simulation [46]. To remove free, non-encapsulated 6BIGOE the NP dispersion was purified by repeated washing steps. However, a certain amount of 6BIGOE might be still attached to the surface or nearby. This is also reflected by the changes of the ZP after 6BIGOE encapsulation, the correlation of DL and ZPs as well as the burst release in the drug release studies.

Several approaches were reported to substitute organic solvents by sustainable solvents for the formulation of PLGA-NP, e.g., PEG 400 [32], methyl propionate [47], or isosorbide dimethyl ether [48]. The main differences between these solvents are the water miscibility which determines the type of preparation technique and therefore, the NP purification method. In case of PEG 400, the viscosity is another important factor for the preparation technique requiring higher process temperature. The PEG 400 method used in our present study was based on a nanoprecipitation technique reported previously [49], which employed PEG 400 to encapsulate lysozyme and bovine serum albumin into PLGA- and PEG-PLGA-NPs obtaining particles sizes < 150 nm with ZP around – 10 mV. The ZP indicated the localization of the proteins on or nearby the NP surface due to their hydrophilicity. Similar to our study, the preparation at 37 °C was necessary to avoid aggregates due to the high viscosity of PEG 400 [49]. In contrast to our study, the NPs were purified by dialysis and not by repeated washing steps with water. A comparable ZP (-3 to -10 mV) was found also by applying water miscible isosorbide dimethyl ether to encapsulate the protein SDF-1α and lysozyme into PLGA/PEG-PLGA-NPs using phase separation [48, 49]. Comparable particle sizes to the results in our study were obtained (200–250 nm). Another approach applying water immiscible methylpropionate was used to encapsulate mestranole into PLGA-NPs with particle sizes in the range of 200–300 nm with an almost neutral ZP [47]. To remove methyl propionate and to harden the NPs, an alkaline hydrolysis technique using NaOH was utilized.

As previously reported [11], high concentrations of 6BIGOE (1 to 3 µM) cause cytotoxic effects in human monocytes, especially over prolonged exposure, i.e., 48 h. Of interest, encapsulation of 6BIGOE into PLGA-NPs, regardless of the formulation method, repressed these detrimental effects of 6BIGOE. Such shielding effects of NPs were recently observed also for doxorubicin- and epoxomicin-loaded PLGA-NPs where encapsulation impaired cytotoxicity of these anti-cancer drugs towards normal cells, but still induced apoptosis in cancer cells upon release of the cargo [50]. Similarly, Eudragit RS 100 affected cell metabolism by binding certain proteins from the culture media and transporting them into cells, thereby increasing cell growth, adhesion and migration [51]. The reduced cytotoxicity of 6BIGOE when encapsulated into PLGA-NPs could be also attributed to lower effective local concentrations of 6BIGOE or delayed release from the NPs, and therefore less off-target effects related to cytotoxicity. Note, however, that 6BIGOE-loaded NPs were equally or even more potent in suppressing GSK-3-mediated cytokine release and COX-2 expression under the same experimental conditions in monocytes, implying that the effective 6BIGOE concentrations inside the cell can be eventually considered quite similar. Possibly, the apparent gradual release of 6BIGOE from the NP and thus delayed cellular availability of 6BIGOE may be causative for the reduced cytotoxicity versus free drug.

We studied the release of 6BIGOE from NP under sink conditions, revealing, in analogy to previous results [29], a biphasic release kinetic with fast and almost complete release of the drug in PBS with 1% Tween 80, pH 7.4, within 8 h independent of the NP preparation method. Rapid hydration and diffusion of drug absorbed or encapsulated close to the surface causes an initial burst release, followed by slower diffusion of the drug through the polymer matrix or water filled pores [52]. For NPs in this size range, drug release is mainly governed by diffusion due to their high surface area to volume ratio and fast water uptake, while degradation of the polymers plays a negligible role [53, 54]. Although for blank NPs prepared by the PEG 400 method a lower crystallinity could be observed, no differences between the methods were evident for 6BIGOE release, as data from DSC analysis revealed comparable T_g_ of 6BIGOE-loaded NPs prepared with the EDE or the PEG 400 method. A comparable fast drug release within hours was also reported by Ali et al. for NP prepared by the PEG 400 method [49], whereas Mansor et al. demonstrated a slow release over days [48]. Moreover, in terms of reduced cytotoxicity, the excellent biocompatibility of the NPs devoid of toxic effects was demonstrated in the dynamic blood flow of a hen’s egg model. This is in accordance with other studies which investigated RG 502 NP in the CAV [29, 30]. Together, the favorable feature of encapsulation into PLGA-NPs provides an additional advantage as higher drug concentrations can be applied without exerting cytotoxic effects. Paired with selective targeting of inflammatory cells, this holds a strong potential for effective directed and specific treatment of inflammatory diseases with reduced adverse effects of 6BIGOE.

Cellular uptake of PLGA-NPs occurs via different endocytic pathways that are widely recognized as major entry process [55, 56]. The preferred uptake of NPs that were prepared by methods using PEG 400 and Cyrene could be affected by remaining solvents, although our previous studies found very low solvent residuals for PEG 400 [29] and Cyrene (< 2.5%) in PLGA-NP using these formulation methods [30]. Notably, PEG-PLGA-NPs are quite similar to NPs formulated with the PEG 400 method [29], implying similar uptake kinetics. Indeed, PEG-PLGA-NPs were found to favorably pass intestinal barriers compared to smaller non-PEGylated NPs [57, 58]. Small amounts of solvent residuals might therefore favor NP uptake. Since NPs prepared by Cyrene and PEG 400 method were overall comparable in their uptake and properties, residual Cyrene may favor NP uptake as well.

Of note, application of empty NPs led to an initial increase of monocyte activities compared to vehicle (0.1% DMSO) control. Nonetheless, encapsulated 6BIGOE could effectively reduce the release of inflammatory mediators as demonstrated for pro-inflammatory cytokine and COX product formation. The activation of monocytes observed with NPs per se could be promoted by the formation of a protein corona [59]. Proteins can attach to the surface of the NPs which leads to recognition by phagocytes, such as monocytes or macrophages that get activated by the stimulus [59], detailed evaluation of the protein corona of PLGA-NPs will be a future task in this respect. This unintended recognition might be circumvented by applying stealth strategies such as PEGylation or introduction of other polymer combinations [59].

6BIGOE is a very potent agent that modulates inflammatory cytokines and LM with significant effects in the one-digit nanomolar range [11]. Therefore, the intention of 6BIGOE encapsulation into NP was not primarily to further increase its potency in the cellular context. Rather, encapsulation may avoid and circumvent undesired off-target interactions of 6BIGOE with outer membranes connected to cytotoxicity, and could also prevent premature drug degradation, which would eventually improve the bioavailability and prolong release kinetics with longer half-lives. This is supported by previous findings with PLGA-NPs that improved oral bioavailability and half-life of hypocrellin A in rats [38]. Along these lines, PLGA-microspheres were shown to increase drug retention in rat joints [60], and PLGA-nanospheres loaded with glucagon demonstrated sustained release and prolonged biological responses in vivo [61]. Interestingly, cytokine release was inhibited by 6BIGOE and 6BIGOE-loaded NPs in a very similar fashion, with even superior potencies of the 6BIGOE-loaded NPs over free drug for inhibition of IL-1β release. In contrast to encapsulated 6BIGOE, free 6BIGOE completely suppressed TNF-α secretion and blocked IL-10 release at higher concentrations, i.e. at 1 µM. The above-mentioned cytotoxic effects of 6BIGOE might be responsible for this but also off-target effects of the free drug on other kinases or receptors are conceivable. For instance, indirubins were shown to inhibit pro-inflammatory cytokine release via MAPK signaling (e.g. p38 MAPK and JNK) in mouse mammary epithelial cells [62], and stimulation of the aryl hydrocarbon receptor, a known target of indirubins, suppressed TNF-α secretion in human microglia [63]. Importantly, encapsulated 6BIGOE retained its ability to enhance the release of the anti-inflammatory cytokine IL-10 (seemingly via GSK-3 suppression [11]) also at higher concentrations, while the free drug reduced IL-10 levels possibly due to off-target effects as discussed above.

Besides cytokines, LMs produced from PUFAs by COX and LOX are crucial regulators of immune responses and the inflammatory process, with either pro-inflammatory (PGs and LTs) or anti-inflammatory/pro-resolving (SPM) properties [43, 64]. In agreement with previous results [11], our LM metabololipidomics approach with LPS-stimulated monocytes revealed selective downregulation of pro-inflammatory COX-derived products by 6BIGOE and 6BIGOE-loaded NPs at low concentrations due to inhibition of COX-2 protein induction without suppression of other LM. When the total amounts of all 5-LOX-derived products were considered, free and encapsulated 6BIGOE did not significantly affect their formation. However, potent inhibition was evident for LTB_4_, which among these 5-LOX products displays potent pro-inflammatory properties [65]. Interestingly, the 6BIGOE derivative 6BIO, was found to be a selective 5-LOX inhibitor via interference with ATP-binding without effects on 12-/15-LOX that lack an ATP-binding site [5]. Similarly, indirubin-3-monoxime (I3MO), devoid of a bromo-substituent, blocked LTB_4_ formation in monocytes and thus prevents migration of vascular smooth muscle cells (VSMC) [66]. Notably, I3MO blocked 12/15-LOX activity in VSMC thereby preventing their proliferation [67]. In our hands, 6BIGOE as free and as encapsulated drug also downregulated the formation of 12-/15-LOX-derived monohydroxylated products in LPS-stimulated monocytes, albeit only at higher concentrations. Future studies will address how 6BIGOE, free and encapsulated, affects functional cellular effects (cell proliferation, chemotaxis, migration, phagocytosis, etc.) in relevant cells that are typically governed by these various LM.

## Conclusion

Taken together, our findings identify PLGA-based delivery systems, prepared by employing encapsulation methods using sustainable solvents, as valuable nanotechnology for improving the therapeutic potential of the highly bioactive small molecule 6BIGOE with, however, various detrimental features as free drug. Thus, encapsulation of this lipophilic molecule with poor solubility, low stability and cytotoxic effects, enables to overcome these unfavorable properties and to shape a favorable pharmacological profile as anti-inflammatory drug. In future experiments, the anti-inflammatory properties of 6BIGOE in NPs as drug delivery system should be comprehensively investigated in in vivo models of inflammation as proof of principle studies to confirm the proposed increase in bioavailability and stability due to encapsulation. Moreover, the influence of the sustainable solvents and the relevant formulation techniques needs further investigation.

## Materials and methods

### Materials

Ethyl acetate (≥ 99.5%, p.a., ISO) was purchased from Carl Roth (Karlsruhe, Germany). Resomer^®^ RG 502 (RG 502, ester terminated, M_W_ 7,000–17,000 g/mol, 50:50 lactide:glycolide ratio) was a kind gift from Boehringer Ingelheim (Ingelheim am Rhein, Germany). Resomer^®^ RG 502H (RG 502H, acid terminated, Mw 7,000–17,000 g/mol) was purchased from Evonik (Essen, Germany). Deuterium-labelled and non-labelled LM standards for UPLC-MS–MS quantification were obtained from Cayman Chemical/Biomol (Hamburg, Germany). 6BIGOE was synthesized as reported before [68]. Solvents and other reagents were purchased from Sigma-Aldrich (Taufkirchen, Germany) unless mentioned otherwise.

### Nanoparticle preparation by emulsion-diffusion-evaporation, PEG 400 and Cyrene method

The NPs were prepared by three different methods, that is, (i) EDE method, (ii) PEG 400 method, and (iii) Cyrene method. 

(i) EDE method. A modified EDE method according to Grune et al. [29] was used as a standard formulation technique. Briefly, 50 mg polymer and 2.5 mg 6BIGOE were dissolved in an organic phase (O-phase), consisting of 4.5 mL ethyl acetate and 0.5 mL 400 g/mol poly(ethylene glycol) (PEG 400, Ph. Eur.). To form an emulsion, a Nemesys syringe pump (Cetoni Automatisierung und Microsysteme, Korbußen, Germany) was used to add the O-phase dropwise (15 µL/s) to a 5 mL aqueous 2% poly(vinyl alcohol) (PVA, 30,000–70,000 g/mol) phase pH 7.4 (W-phase), followed by 3 h magnetic stirring. Subsequently, the emulsion was homogenized by an Ultra-Turrax (IKA-Werke, Staufen, Germany) homogenizer (24,000 rpm, 15 min). After dilution with deionized water the emulsion was stirred for further 16 h at room temperature to evaporate the ethyl acetate. The NP dispersion was washed thrice with 50 mL water by centrifugation (22,000 × *g*, 20 min). The NP yield was calculated by weighting as the ratio of NPs (mg) to the total weight of polymer and 6BIGOE (mg) after lyophilization for 72 h (-30 °C, 0.06 bar; Lyovac GT2, Finn-Aqua Santasalo-Sohlberg, Hürth, Germany).

(ii) PEG 400 method. A preparation method using PEG 400 was used to formulate 6BIGOE NPs according to Grune et al. [29]. In brief, 2.5 mg 6BIGOE and 50 mg polymer were dissolved in 3 mL PEG 400 and added dropwise using the Nemesys syringe pump (15 µL/s) to a 15 mL 2% PVA containing W-phase under magnetic stirring at 650 rpm. The preparation was stirred for 5 h at 37 °C, before collecting, washing and lyophilization as described above.

(iii) Cyrene method. Additionally, NPs were prepared using Cyrene according to Grune et al. [30]. Ten mg polymer and 0.5 mg 6BIGOE were dissolved in 1 mL Cyrene. This solution was covered with 5 mL W-phase and sonicated for 30 s at a cycle of 100% (Sonopuls HD200, Bandelin electronic, Germany). Subsequently, the NP dispersion was stirred (magnetic stirring) for 2 h before collecting, washing and lyophilization as described above.

For visualization of the NPs during cell uptake experiments, fluorescein-labeled PLGA-NPs with and without 6BIGOE were prepared using the same methods as described above. Therefore, fluorescein was covalently coupled to Resomer^®^ RG 502H (Additional file 1: SI1) and NPs were formulated with a mixture of 1:4 FITC-PLGA and RG 502.

### Laser light scattering techniques

A Zetasizer Nano ZS (Malvern Instruments, Herrenberg, Germany) equipped with the Zetasizer v7.12 software (Malvern Instruments Ltd, Worcestershire, UK) was used to analyze the HD, PDI and ZP of the NPs. Photon correlation spectroscopy was used to measure HD and PDI of an aqueous NP dispersion in ZEN 0112 cuvettes (Brand, Wertheim, Germany) at 633 nm and 25 °C under a scattering angle of 173 °C. Five runs per sample were used to calculate the mean values ± SD of at least four independent NP preparations. To determine the ZP the electrophoretic mobility was measured in DTS 1070 capillary cells (Malvern Instruments) at 25 °C. A refractive index of 1.33 and a viscosity of 0.88 mPa‧s were used for calculations. At least four independent NP preparations with three runs per sample were analyzed and represented as mean values ± SD (intensity weighted distribution).

### Atomic force microscopy imaging (AFM)

A dispersion of NP (10 µL) was drop cast on freshly cleaned mica substrates and air-dried. To image the blank and loaded NPs obtained from EDE, Cyrene or PEG 400 method, a Dimension 3100 (Digital Instruments, Veeco, Santa Barbara, CA) equipped with a Nanoscope IV controller as well as a JPK-Nanowizard (JPK BioAFM, Bruker Nano, Berlin, Germany) was used. Standard tapping mode silicon cantilevers from Bruker (model RTESPA 300, Bruker, Santa Barbara, CA) with a resonance frequency around 300 kHz in air, a spring constant in the range of 20 to 80 N/m, and a tip radius of less than 10 nm (typically 7 nm) were used for height imaging.

### 6BIGOE quantification by UV/Vis spectrophotometry

Quantification of 6BIGOE was performed by UV/Vis spectrophotometry. Therefore, 6BIGOE-loaded NPs were dissolved in DMSO and analyzed by Tecan Spark 10 M (Tecan Group, Männedorf, Switzerland) at 520 nm. The calibration solutions were prepared with DMSO ranging between 0.156–25 µg/mL (linearity of response: r^2^ = 0.9998; limit of detection (LOD) = 0.41 µg/mL; limit of quantification (LOQ) = 1.36 µg/mL). The DL (%) was calculated as the ratio of encapsulated drug (mg) per 100 mg NPs by weighting and presented as mean ± SD from at least four independent batches.

### In vitro drug release studies

The in vitro drug release of 6BIGOE from NPs was investigated under sink conditions in 10 mL PBS with 1% Tween 80 (m/m) (both Carl Roth, Karlsruhe, Germany) pH 7.4 in a heated incubator (37° C Bachofer, Weilheim an der Teck, Germany) under horizontal rotation. Samples were withdrawn at 0.5, 1, 2, 4, 8, 24, 48, 72 and 168 h, followed by centrifugation with 25,000 × g for 5 min. The supernatant was analyzed by UV/Vis spectroscopy as described above. Data were reported as mean ± SD of three independent batches and fitted by a two-phase exponential model (function: ExpDec2) (r^2^ = 0.999). To ensure sink conditions, the solubility of 6BIGOE in the release medium was determined from the supernatant of a saturated solution after centrifugation (30,000 × g, 15 min) and quantified by UV/Vis as described above.

### Differential scanning calorimetry

Differential scanning calorimetry analysis of the NPs was performed using a DSC 250 (TA Instruments, New Castle, USA). The NP samples were heated in Tzero aluminium pans from -10 to 190 °C applying a heating rate of 10 °C/min under N_2_ atmosphere. Calibration was carried out with indium according to the manufacturer’s specifications. Each sample was measured in two heating–cooling cycles to evaluate the phase transition enthalpy (J/g; first run) and T_g_ (second run), respectively. TRIOS v4.3.1.39215 software (TA Instruments) was used for data analysis.

### Blood cell isolation and monocyte incubation

Leukocyte concentrates from freshly withdrawn peripheral blood from healthy human volunteers (age 18 – 65 years) were obtained from the Institute of Transfusion Medicine at the University Hospital Jena (Jena, Germany). All protocols for experiments involving human blood cells were approved by the ethical commission (approval no. 5050–01/17) of the Friedrich Schiller University Jena (Jena, Germany). All methods were performed in accordance to the relevant guidelines and regulations. Briefly, peripheral blood mononuclear cells were separated using dextran sedimentation, followed by centrifugation on lymphocyte separation medium (Histopaque-1077). Isolation of monocytes from the peripheral blood mononuclear cells fraction was achieved by adherence to culture flasks (Greiner, Nuertingen, Germany) for 1 h at 37 °C and 5% CO_2_ in RPMI 1640 medium supplemented with 10% (v/v) heat-inactivated fetal calf serum, 2 mM L-glutamine (Biochrom/Merck, Berlin, Germany), 100 U/mL penicillin, and 100 µg/mL streptomycin. Routinely, monocytes were then harvested and seeded in RPMI 1640 containing 5% (v/v) heat-inactivated fetal calf serum, 2 mM L-glutamine, 100 U/mL penicillin, and 100 µg/mL streptomycin (monocyte medium) for further experiments. Unless stated otherwise, vehicle (0.1% (v/v) DMSO for 6BIGOE or equivalent amount of blank NP dispersion), 6BIGOE or 6BIGOE-loaded NPs were added 3 h prior to stimulation with 100 ng/mL LPS (Sigma-Aldrich) for 18 h at 37 °C and 5% CO_2_.

### Determination of cell viability

The viability of monocytes was determined by MTT assay as described [69]. Briefly, monocytes were treated with vehicle as negative control, that is, 0.5% (v/v) DMSO for 6BIGOE or PBS 0.1% for blank NP and 6BIGOE-loaded NPs, equivalent to the amounts of blank NP dispersion or 6BIGOE-loaded NPs, and incubated for 24 or 48 h at 37 °C and 5% CO_2_. Staurosporine (1 µM), an inducer of cell death, was used as positive control. MTT solution was added, cells were incubated for further 4 h, lysed in buffer containing 10% (w/v) SDS, and the absorbance was measured at 570 nm (Multiskan Spectrum, Thermo Fisher, Waltham, MA).

### Ex ovo shell-less hen’s egg model

An ex ovo shell-less hen’s egg model was used to investigate toxic reactions of the NPs according to Schlenk et al. [40]. Fertilized white chicken eggs from a local supplier were incubated in an incubator at 37 °C for 72 h followed by transfer of the content into petri dishes containing Ringer’s solution pH 7.0. Only intact eggs with a well-developed CAV at stages 14–17 according to Hamburger and Hamilton [70] were used after 24 h of further incubation. Two microliters of the NP dispersions were injected by a microinjector (Sutter Instrument Company, Novato, USA) and a Hamilton syringe into the vitelline vein using a concentration of 3 µM, calculated on the 6BIGOE DL of the NPs. According to the recommendations of the Interagency Coordinating Committee on the Validation of Alternative Method (ICCVAM) [71], 0.9% sodium chloride solution (negative control, Carl Roth), deionized water (solvent control) and 25 mg/mL 25,000 g/mol branched PEI solution in water (positive control, kindly provided by BASF SE, Ludwigshafen, Germany) were used as negative, solvent and positive control, respectively. Eggs were inspected under a stereomicroscope after 0, 1, 2, 4, 8 and 24 h for vascular lysis, hemorrhage, aggregation and cardiac arrest. The experiment was run with five eggs per NP sample and repeated independently once. Historical lab values are a collection of the effects from the negative, solvent, and positive controls of former experiments.

### Uptake of fluorescein-labeled NPs in monocytes

Uptake of fluorophore-labeled NPs by human monocytes was evaluated using flow cytometry and microscopy. Monocytes (2 × 10^6^) were incubated with 6BIGOE-loaded fluorescein-labeled NPs at a concentration of 15 µg NPs per mL medium for 30 min, 3 h or 6 h at 37 °C and 5% CO_2_. Cells were detached using PBS pH 7.4 containing 5 mM EDTA and 0.1% sodium azide supplemented with 0.4% lidocaine and transferred to a clean tube. Dead cells were stained using Zombie Aqua™ (BioLegend, San Diego, CA, USA) before blocking of unspecific antibody binding with mouse serum (10 min, 4 °C). Subsequently, cells were stained in flow cytometry buffer (PBS containing 0.5% BSA, 5 mM EDTA and 0.1% sodium azide) with APC anti-human CD14 antibody (clone HCD14, BioLegend) for 20 min on ice to identify monocytes. Monocytes containing fluorescein-labeled NPs were analyzed using a BD LSR Fortessa (BD Bioscience, Heidelberg, Germany). For flow cytometric analysis, 640 nm excitation in combination with 670/14 bandpass filter was used to detect APC-CD14^+^ monocytes and 488 nm excitation with 530/30 bandpass filter was used to detect fluorescein-labeled NPs. Obtained data were analyzed using FlowJo X Software (BD Bioscience).

For analysis of cellular NP uptake using fluorescence microscopy, human monocytes (0.8 × 10^6^) were seeded onto glass coverslips and incubated with 6BIGOE-loaded fluorescein-labeled NPs at a concentration of 15 µg NP per mL medium for 3 h at 37 °C and 5% CO_2_. Cells were fixed applying 4% paraformaldehyd solution for 20 min, then cells were washed with PBS pH 7.4 and mounted onto glass slides using ProLong™ Diamond Antifade Mountant with DAPI (Fisher Scientific, Schwerte, Germany), which simultaneously served to stain nuclear DNA. Subsequently, cells and fluorophore-labeled NPs were visualized with a Zeiss Axio Observer Z1 microscope and a Plan-Apochromat 40x/1.3 Oil DIC M27 objective (Carl Zeiss, Jena, Germany). Images were captured with an AxioCam MRm camera, cut and exported using AxioVision 4.8.2.0 software (Carl Zeiss).

### Determination of extracellular cytokine levels

Monocytes (10^6^/mL) were treated with vehicle, 6BIGOE or 6BIGOE-loaded NPs at the indicated concentrations and time points and stimulated with 100 ng/mL LPS as described. Subsequently, extracellular cytokine levels were measured in cell-free supernatants using enzyme-linked immunosorbent assay (ELISA), according to manufacturer’s instructions (R&D Systems, Minneapolis, Minnesota).

### SDS-PAGE and Western blot

To obtain cell lysates, monocytes (2 × 10^6^) were resuspended in lysis buffer containing 1% (v/v) NP-40 (AppliChem, Darmstadt, Germany), 1 mM sodium orthovanadate (AppliChem), 10 mM sodium fluoride (AppliChem), 5 mM sodium pyrophosphate (Sigma-Aldrich), 25 mM β-glycerophosphate (Sigma-Aldrich), 5 mM EDTA (AppliChem), 25 µM leupeptin (Sigma-Aldrich), 3 µM soybean trypsin inhibitor (Sigma-Aldrich) and 1 mM phenylmethanesulfonyl fluoride (Sigma-Aldrich), transferred to clean tubes and lysed on ice for 20 min with occasional vortexing. After centrifugation (20,000 × *g*, 5 min, 4 °C), total protein content of cell-free supernatants was determined using the DC protein assay kit (Bio-Rad Laboratories GmbH, Munich, Germany). Samples were then mixed with 1 × SDS/PAGE sample loading buffer containing 125 mM Tris–HCl (pH 6.5), 25% (m/v) sucrose, 5% SDS (m/v), 0.25% (m/v) bromophenol blue, and 10% (v/v) β-mercaptoethanol, and protein denaturation was achieved by boiling for 5 min at 95 °C.

Aliquots (10 µg total protein) were separated on 10% polyacrylamide gels. Proteins were subsequently transferred onto nitrocellulose membranes (Amersham Protran supported 0.45 µm nitrocellulose; GE Healthcare) for protein detection which was achieved by incubation with the following primary antibodies: rabbit monoclonal anti-COX-2, 1:500 (12282S, Cell Signaling, Danvers, MA), mouse monoclonal anti-β-actin, 1:1000 (3700S, Cell Signaling). Immunoreactive band were then stained with IRDye 800 goat anti-rabbit IgG, 1:15,000 (926–32,211, LI-COR Biotechnology, Cambridge, UK) and IRDye 680LT goat anti-mouse IgG, 1:40,000 (926–68,020, LI-COR Biotechnology), and visualized using an Odyssey infrared imager (LI-COR Biosciences). Data from densitometric analysis were background-corrected.

### Sample preparation for UPLC-MS–MS analysis

Cell-free supernatants obtained from cell culture experiments were mixed in a ratio of 1:2 (v/v) with ice-cold methanol (Fisher Chemical, Schwerte, Germany) containing 10 µL of deuterium-labelled standards as internal reference. Deuterated standards include 200 nM d_8_-5*S*-hydroxyeicosatetraenoic acid (HETE), d_4_-LTB_4_, d_5_-lipoxin A_4_, d_5_-resolvin D2, d_4_-PGE_2_ and 10 µM d_8_-arachidonic acid. Before proceeding with purification steps, proteins were precipitated at -20 °C overnight. After centrifugation (1200 × *g*, 10 min, 4 °C), the supernatant was collected and acidified by addition of 7 mL Milli-Q water (pH 3.5) for solid phase extraction. C18 solid phase cartridges (Waters, Eschborn, Germany) were washed with 6 mL methanol and equilibrated with 2 mL Milli-Q water before sample addition. Subsequently, columns were washed with 6 mL of Milli-Q water to return to neutral pH ~ 7.0. Following another washing step with 6 mL of *n*-hexane (Fisher Chemical), eicosanoids, docosanoids and PUFAs were eluted with 6 mL of methyl formate (Acros Organics, Schwerte, Germany). Samples were evaporated until dryness with a moderate stream of nitrogen (TurboVap LV, Biotage; Uppsala, Sweden) and resuspended in 100 µL of a mixture of methanol and Milli-Q water (50:50, v/v).

### UPLC-MS–MS-assisted profiling of lipid mediators

The LM profiles were acquired via UPLC-MS–MS following an established protocol [44]. Briefly, eicosanoids, docosanoids and PUFAs were separated utilizing an Acquity™ UPLC system (Waters) equipped with an Acquity™ UPLC BEH C18 column (Waters; 1.7 µm, 2.1 × 100 mm). Column temperature was set to 50 °C and flow rate to 0.3 mL/min. Analytes were eluted using a gradient method with a mobile phase consisting of methanol, water and acetic acid in a ratio of 42:58:0.01 (v/v/v), which was ramped up to 86:14:0.01 (v/v/v) over 12.5 min and finally set at 98:2:0.01 (v/v/v) for 3 min. Detection of analytes was achieved using a QTRAP 5500 mass spectrometer (AB Sciex, Darmstadt, Germany) equipped with electrospray ionization, operating in negative ionization mode using scheduled multiple reaction monitoring (MRM) combined with data-dependent acquisition. The scheduled MRM window was set to 60 s, optimized LM parameters (collision energy, entrance potential, declustering potential, collision cell exit potential) were adopted and curtain gas pressure set at 35 psi. External standards (Cayman Chemicals) were used to confirm retention time and at least six diagnostic ions for each LM, as previously reported [72]. Detected analytes were then quantified via linear calibration curves, which were obtained for each analyte with *r*^2^ values of 0.98–0.99, and limit of detection determined [72]. To account for deviations due to sample preparation and handling, concentration of all detected analytes was normalized upon internal deuterium-labelled standards.

### Statistical analysis

Data are expressed as means ± SD or means + SEM of *n* observations, as indicated, where *n* represents the number of independent experiments with separate donors performed on different days. Analyses of data were conducted using GraphPad Prism software (Version 9.1.2, San Diego, CA). Normal or lognormal distribution of data was determined using Shapiro–Wilk test, and data log-transformed for further analysis as indicated. Comparison of two groups was then conducted using unpaired, two-tailed Student’s *t*-test as indicated. For multiple comparisons, one-way-ANOVA with or without repeated measurements with Geisser-Greenhouse correction and either Holm-Šídák or Dunnett’s post-hoc test was applied as indicated. The NP characteristics were analyzed by one-way ANOVA followed by Tukey post-hoc test using Origin 2019 (version 9.6.0.172). The criterion for statistical significance is *p* < 0.05, statistical significance is indicated as: *p < 0.05, **p < 0.01, ***p < 0.001, ****p < 0.0001, ns = not significant, unless stated otherwise.

## Supplementary Information

**Additional file 1.** Supplemental information, figures and tables.

## Data Availability

The datasets used and/or analyzed during the current study are available from the corresponding authors on reasonable request.

## Abbreviations

AFM: Atomic force microscopy
6BIGOE: 6-Bromoindirubin-3'-glycerol-oxime ether
6-BIO: 6-Bromoindirubin-3'-oxime
COX: Cyclooxygenase
DMSO: Dimethyl sulfoxide
DL: Drug load
EDE: Emulsion-diffusion-evaporation
DSC: Differential scanning calorimetry
EDTA: Ethylenediaminetetraacetic acid
ELISA: Enzyme-linked immunosorbent assay
FITC: Fluorescein-5-isothiocyanate
GSK: Glycogen synthase kinase
HD: Hydrodynamic diameter
HPLC: High performance liquid chromatography
IL: Interleukin
LM: Lipid mediator
LOD: Limit of detection
LOQ: Limit of quantification
LOX: Lipoxygenase
LPS: Lipopolysaccharide
NP: Nanoparticles
PAGE: Polyacrylamide gel electrophoresis
PBS: Phosphate-buffered saline
PEG 400: 400 g/mol poly(ethylene glycol)
PEI: Poly(ethylene imine)
PLGA: Poly(d,l-lactide-co-glycolide)
PUFA: Polyunsaturated fatty acid
PVA: Poly(vinyl alcohol)
SD: Standard deviation
SDS: Sodium dodecyl sulfate
SEM: Standard error of the mean
STSP: Staurosporine
T_g_: Glass transition temperature
TNF: Tumor necrosis factor
UPLC-MS–MS: Ultra-performance liquid chromatography-tandem mass spectrometry
UV/Vis: Ultraviolet–visible spectrophotometry
ZP: Zeta potential
