# Supplementary material for: Drug delivery of 6-bromoindirubin-3’-glycerol-oxime ether employing poly(d,l-lactide-co-glycolide)-based nanoencapsulation techniques with sustainable solvents
Source: J Nanobiotechnology. 2022 Jan 4;20:5. doi: 10.1186/s12951-021-01179-7 (PMC8725458; doi:10.1186/s12951-021-01179-7)
Supplement: Supplementary file 1 — Additional file 1. Supplemental information, figures and tables. [file 12951_2021_1179_MOESM1_ESM.docx]

Supplementary Information

**Drug delivery of 6-bromoindirubin-3’-glycerol-oxime ether employing poly(d,l-lactide-co-glycolide)-based nanoencapsulation techniques with sustainable solvents**

Anna Czapka^a,§^, Christian Grune^b,§^, Patrick Schädel^a^, Vivien Bachmann^a^, Karl Scheuer^c^, Michael Dirauf^d,f^, Christine Weber^d,f^, Alexios-Leandros Skaltsounis^e^, Klaus D. Jandt^c,f^, Ulrich S. Schubert^d,f^, Dagmar Fischer^b,f,g,*^, and Oliver Werz^a,f,*^

^a^ Department of Pharmaceutical/Medicinal Chemistry, Institute of Pharmacy, Friedrich Schiller University Jena, Philosophenweg 14, 07743 Jena, Germany.

^b^ Pharmaceutical Technology and Biopharmacy, Institute of Pharmacy, Friedrich Schiller University Jena, Lessingstraße 8, 07743 Jena, Germany.

^c^ Chair of Materials Science (CMS), Otto Schott Institute of Materials Research, Faculty of Physics and Astronomy, Friedrich Schiller University Jena, Löbdergraben 32, 07743 Jena, Germany.

^d^ Laboratory of Organic and Macromolecular Chemistry (IOMC), Friedrich Schiller University Jena, Humboldtstraße 10, 07743 Jena, Germany

^e^ Department of Pharmacy, Division of Pharmacognosy and Natural Products Chemistry, University of Athens, Panepistimiopolis Zografou, GR-15771 Athens, Greece.

^f^ Jena Center for Soft Matter (JCSM), Friedrich Schiller University Jena, Philosophenweg 7, 07743 Jena, Germany.

^g^ Division of Pharmaceutical Technology, Department for Chemistry and Pharmacy, Friedrich‑Alexander-University Erlangen‑Nürnberg, Cauerstrasse 4, 91058 Erlangen, Germany.

**Suppl. Information SI1.** **Synthesis of FITC-PLGA**.

*Reagents*

Fluorescein-5-isothiocyanate (FITC, 90% pure, Acros Organics), Amberlyst A21 (Alfa Aesar), trifluoroacetic acid (TFA, HPLC grade 99.5+%, ABCR), Resomer RG502H (PLGA, acid terminated, Mw 7,000 – 17,000 g/mol, Evonik), triethylamine (anhydrous ≥ 99%, Sigma Aldrich), *N*,*N*‘-dicyclohexylcarbodiimide (DCC, 99%, Sigma Aldrich), 4-dimethylaminopyridine (DMAP, 99%, Sigma Aldrich), *N*-Boc-ethylenediamine (≥ 98%, Sigma Aldrich) and were used as received. Dichloromethane and *N*,*N*-dimethylformamide (DMF) were dried in solvent purification system (Pure solv EN, InnovativeTechnology).

*Instrumentation*

All nuclear magnetic resonance (NMR) spectra were measured on a 300 MHz spectrometer from Bruker equipped with an Avance I console and a dual ^1^H and ^13^C sample head. The shifts are given in ppm using the residual non-deuterated solvent for chemical shift referencing.

Size exclusion chromatography (SEC) was measured on a system from Agilent equipped with a PSS degasser, a G1310A pump, a G1329A autosampler and a techlab oven (40 °C). The signals were detected using a G7162A RI detector. 0.21 m% LiCl in *N*,*N*-dimethylacetamide (DMAc) was applied as eluent. A column set consisting of a PSS GRAM 30 Å and 1,000 Å (10 µm particle size) placed in series was utilized for separation. The flow rate was set constant as 1 mL min^-1^ and the molar masses were estimated using PMMA standards (ca. 400 to 1,000,000 g mol^-1^) from PSS.

*Synthesis*


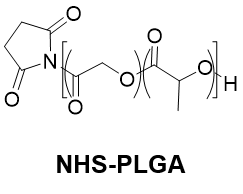


**NHS-PLGA**: The NHS functionalized PLGA was synthesized applying a literature-known procedure (1). In detail, PLGA Resomer RG502H (2515 mg, 0.24 mmol, 1 eq.) and *N*-hydroxy succinimide (NHS, 100 mg, 0.86 mmol, 3.6 eq.) were suspended in 20 mL dry dichloromethane. *N*,*N*’-dicyclohexylcarbodiimide (DCC, 180 mg, 0.87 mmol, 3.6 eq.) was dissolved in 2 mL dry dichloromethane and subsequently added to the PLGA solution. A solution containing 4-dimethylaminopyridin (DMAP, 5.9 mg, 0.05 mmol, 0.2 eq.) in dichloromethane was added. The mixture was stirred at ambient temperature overnight. Subsequent to filtration, the solvent was removed under reduced pressure. The remaining viscous oil was re-dissolved in a small amount of dichloromethane, precipitated from – 26 °C diethyl ether and dried *in vacuo*, yielding NHS-PLGA as a colourless solid.

Yield: 2490 mg (98%). SEC (DMAc, 0.21 m% LiCl, RI-detection, PMMA-calibration): M_n_ = 14,300 g mol^-1^, Đ = 2.14, ^1^H-NMR (300 MHz, DMSO-d_6_): *δ* [ppm] = 1.34 – 1.64 (br, 123H, CH_3_), 2.82 (br, 4H, CH_2_ NHS), 4.69 – 5.05 (br, 78H, CH_2_), 5.05 – 5.36 (br, 41H, CH).


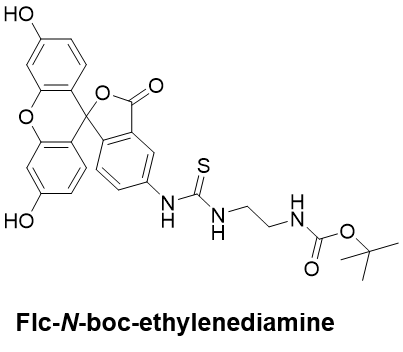


**Flc-*N*-boc-ethylenediamine**: The compound was synthesized according to a literature-known procedure with slight adjustments (2). Fluorescein-5-isothiocyanate (FITC, 325 mg, 0.83 mmol, 1 eq.) and *N*-Boc-ethylenediamine (154 mg, 0.96 mmol, 1.15 eq.) were dissolved in 4 mL dry DMF. Subsequently, triethylamine (62 mg, 0.61 mmol, 0.7 eq.) was added and the vial was sealed under inert atmosphere. The mixture was stirred at ambient temperatures overnight. Subsequently, an aliquot was removed and analysed by thin layer chromatography using pure ethyl acetate as mobile phase (R_f_ = 0.7). The product was isolated by flash silica gel chromatography using ethyl acetate as a mobile phase. The product containing fractions were combined and the volatiles were removed under reduced pressure.

Yield: 335 mg (73%). ^1^H-NMR (300 MHz, DMSO-d_6_): *δ* [ppm] = 1.39 (s, 9H, Boc), 3.17 (m, 2H, CH_2_), 3.57 (m, 2H, CH_2_), 6.47 – 6.72 (m, 6H, CH_Ar_), 6.97 (m, 1H, NH), 7.20 (d, 1H, CH_Ar_), 7.74 (d, 1H, CH_Ar_), 8.07 (s, 1H, NH), 8.21 (s, 1H, CH_Ar_) 10.00 (s, 1H, NH), 10.13 (s, 2H, OH).


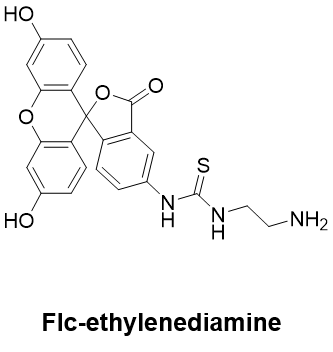


**Flc-ethylenediamine**: The compound was synthesized according to literature known procedures with slight adjustments (2,3). Flc-*N*-Boc-ethylenediamine (300 mg, 0.5 mmol, 1 eq.) was dissolved in 15 mL of a mixture of dichloromethane/trifluoroacetic acid (1/1, v/v) and stirred at room temperature for one hour. The solvent mixture was removed by cold distillation. The residue was re-dissolved in ethyl acetate and filtered through a syringe containing amberlyst A21 ion exchance resin. The volatiles were removed and the remaining viscous oil crystallized overnight. The product was characterized by ^1^H NMR spectroscopy and used without further purification.

Yield: 292 mg. ^1^H NMR (300 MHz, DMSO-d_6_): *δ* [ppm] = 3.07 (m, 2H, CH_2_), 3.78 (m, 2H, CH_2_), 6.44 – 6.75 (m, 6H, CH_Ar_), 7.22 (d, 1H, CH_Ar_), 7.75 (d, 1H, CH_Ar_), 7.82 (3H, NH_3_^+^), 8.20 (s, 1H, CH_Ar_), 8.30 (t, 1H, NH), 9.44 – 10.70 (br, 2H, OH), 10.32 (s, 1H, NH).


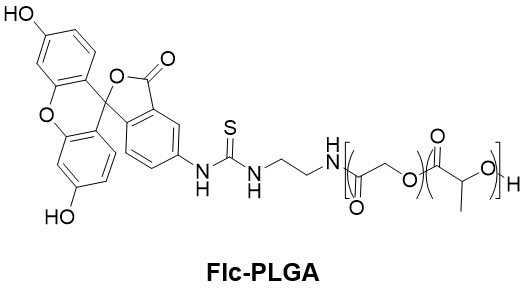


**Flc-PLGA**: NHS-PLGA (1531 mg, 0.14 mmol, 1 eq.) was dissolved in 12 mL dry DMF. A solution of Flc-ethylenediamine (70 mg, 0.16 mmol, 1.1 eq.) in 2 mL dry DMF was added. Subsequently, triethylamine (30 µL, 0.21 mmol, 1.5 eq.) was added *via* syringe. The mixture was stirred at ambient temperatures for 18 h. The solvent was evaporated, the residue was dissolved in ca. 300 mL dichloromethane and washed with deionized water twice (â 300 mL). The aqueous phase was extracted with 250 mL dichloromethane. The combined organic phases were dried over Na_2_SO_4_, filtered and the solvent was evaporated under reduced pressure. The residue was precipitated from –26 °C diethyl ether. This process was repeated once and the polymer was additionally precipitated from –26 °C methanol. Finally, the polymer was dissolved in DMSO, dialyzed against deionized water (molecular weight cut-off 3,5 kDa) and lyophilized, yielding Flc-PLGA as a yellowish solid.

Yield: 501 mg (31%). SEC (DMAc, 0.21 m% LiCl, RI-detection, PMMA-calibration): M_n_ = 17,300 g mol^-1^, Đ = 2.07, ^1^H-NMR (300 MHz, DMSO-d_6_): *δ* [ppm] = 1.08 – 1.72 (br, 123H, CH_3_), 4.52 – 5.07 (br, 78H, CH_2_), 5.07 – 5.65 (br, 41H, CH), 6.47 – 6.75 (br, CH_Ar_), 7.20 (br, CH_Ar_), 7.70 (br, CH_Ar_), 7.97 – 8.30 (br, CH_Ar_ and NH), 9.94 – 10.21 (br, OH and NH).

**References**

1. Deng C, Tian H, Zhang P, Sun J, Chen X, Xiabin J. Synthesis and characterization of RGD peptide grafted poly(ethylene glycol)-b-poly(l-lactide)-b-poly(l-glutamic acid) triblock copolymer. Biomacromolecules. 2006 Feb;7(2):590–6.

2. Hananya N, Boock AE, Bauer CR, Satchi-Fainaro R, Shabat D. Remarkable enhancement of chemiluminescent signal by dioxetane–fluorophore conjugates: turn-on chemiluminescence probes with color modulation for sensing and imaging. J Am Chem Soc. 2016 Oct 12;138(40):13438–46.

3. Srinivasan N, Yurek-George A, Ganesan A. Rapid deprotection of N-Boc amines by TFA combined with freebase generation using basic ion-exchange resins. Mol Divers. 2005 Nov;9(4):291–3.

**Figures**


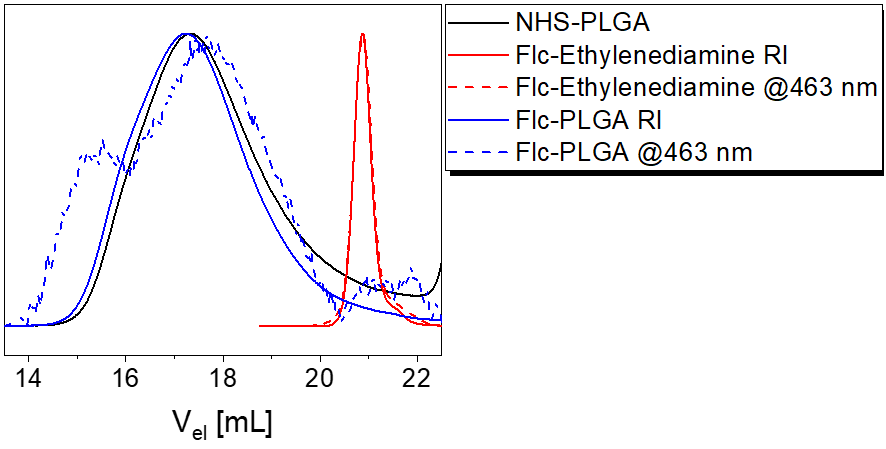


**Suppl. Figure S1**. Overlay of the SEC elugrams (DMAc, 0.21m% LiCl) of Flc-ethylenediamine (red), NHS-PLGA (black) and Flc-PLGA (blue). The solid lines represent the refractive index signals whereas the dashed lines represent the absorbance at a wavelength of 463 nm.


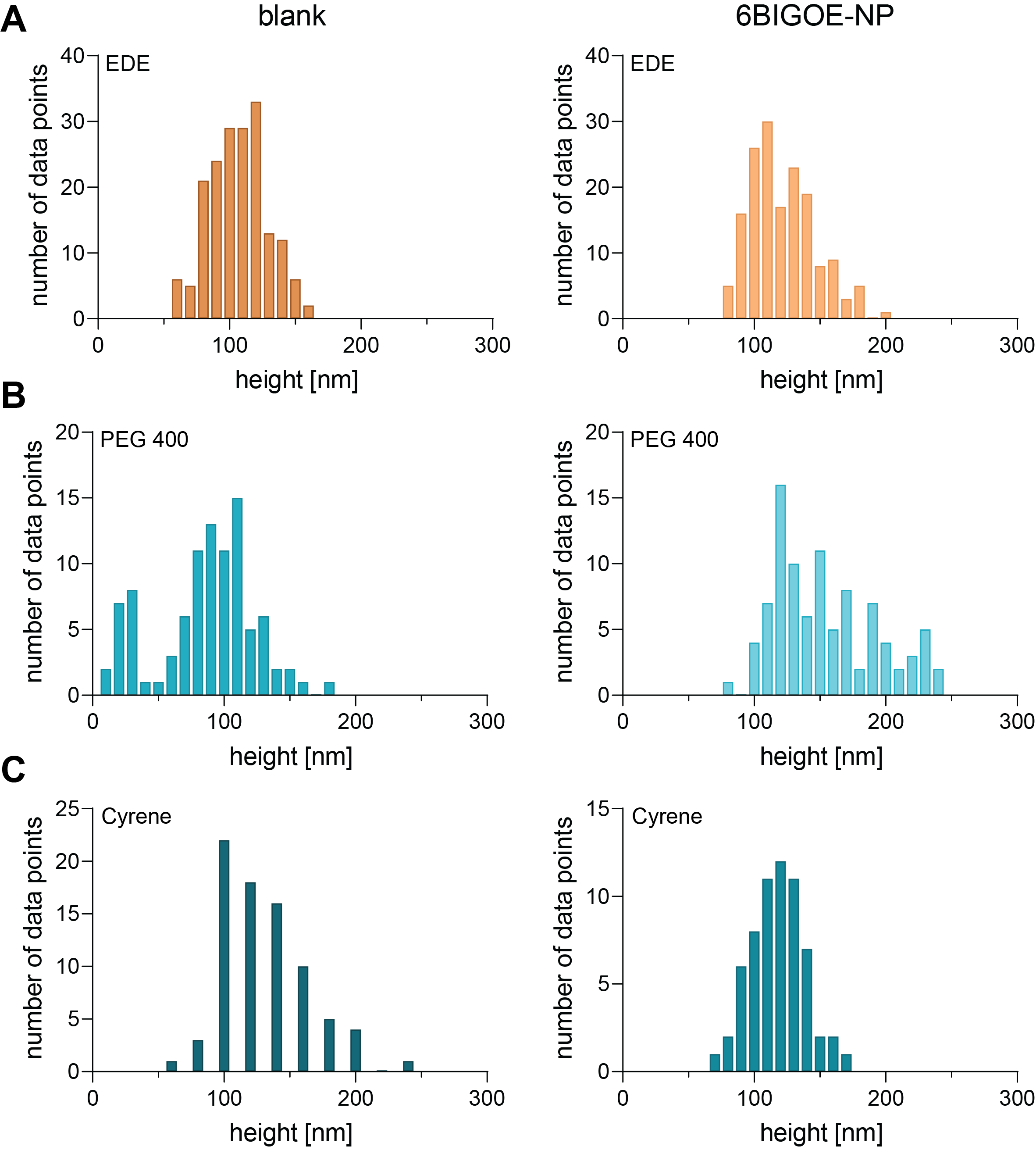


**Suppl. Figure S2: Evaluation of NP height distribution.** NP heights determined from images obtained by atomic force microscopy (AFM) of the blank and 6BIGOE‑loaded nanoparticles prepared by (**A**) emulsion-diffusion-evaporation (EDE), (**B**) PEG 400 method or (**C**) Cyrene method showing a monomodal size distribution. Height distributions of 63 to 180 data points are displayed as histograms.

**
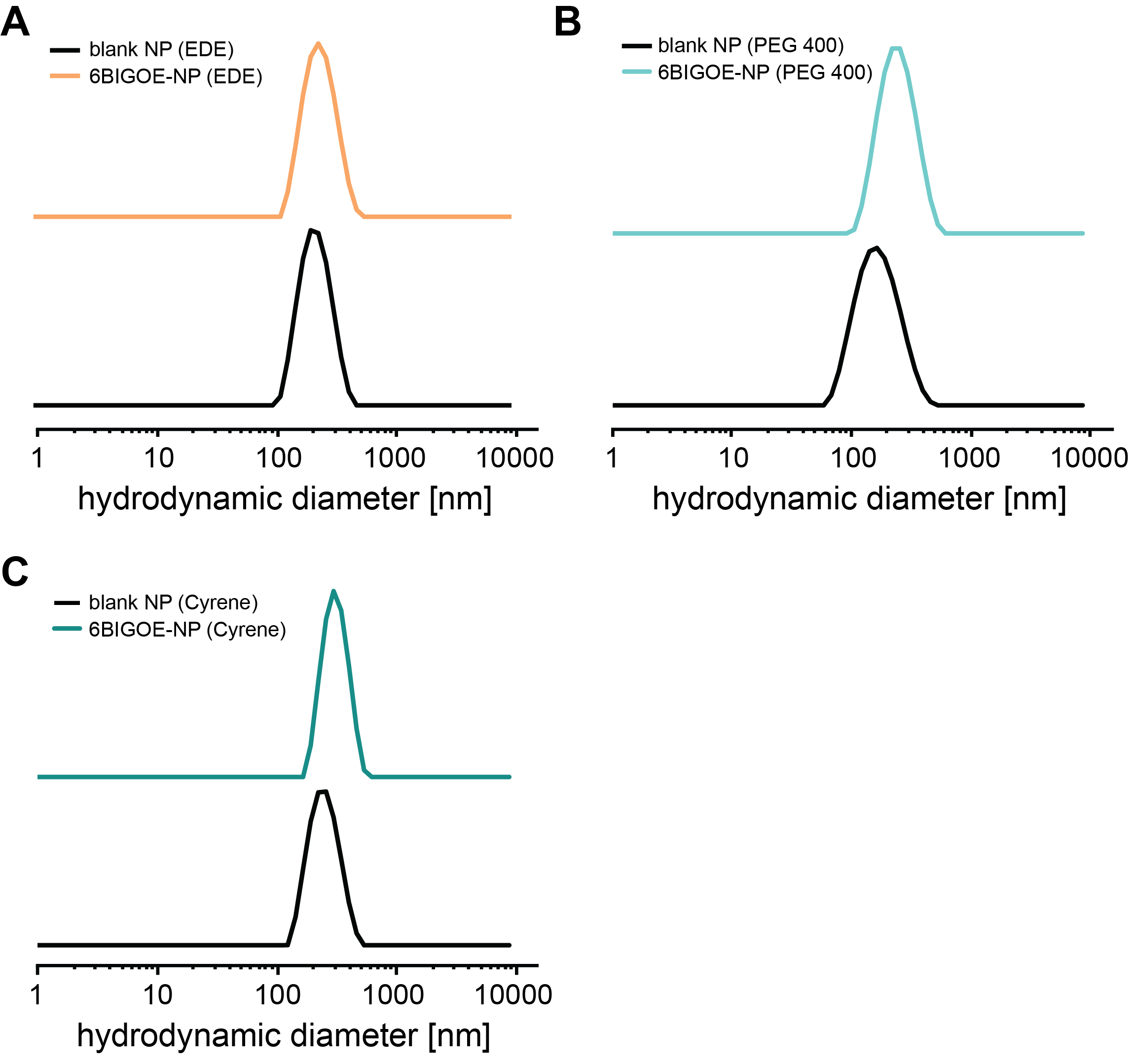
**

**Suppl. Figure S3: Characterization of NP size distribution.** Exemplary intensity weighted size distributions obtained by dynamic light scattering measurements of the blank and 6BIGOE‑loaded nanoparticles prepared by (**A**) emulsion-diffusion-evaporation (EDE), (**B**) PEG 400 method or (**C**) Cyrene method showing a monomodal size distribution.

**Suppl. Figure S4: Assessment of the cytotoxic potential of blank NPs**. Cell viability of human monocytes was evaluated by MTT assay. Cells were treated for 24 h (upper panel) or 48 h (lower panel) with vehicle (0.1% PBS (v/v)), or with blank NPs prepared by emulsion-diffusion-evaporation (EDE), Cyrene, or PEG 400 method in amounts equivalent to the indicated concentration of 6BIGOE‑loaded NPs, at 37 °C, and MTT assay was performed by measuring the absorbance at 570 nm. Values are means + SEM; expressed as percentage of control (vehicle = 100%), n = 4 separate donors. Statistical analysis was performed applying repeated-measurement one-way ANOVA with Geisser-Greenhouse correction and Holm-Šídák multiple comparisons test, testing treatments against vehicle (0.1% PBS (v/v)). Data was log-transformed prior to analysis for NP blanks at both time points.

**Suppl. Figure S5. Influence of 6BIGOE on lipid mediator formation in human monocytes.** Human monocytes (10^6^/mL) were pretreated with vehicle (0.1% DMSO (v/v)), or 6BIGOE at 0.3 µM for 3 h before stimulation with 100 ng/mL LPS for 18 h at 37 °C. Unstimulated control was neither treated nor stimulated. Lipid mediator formation was measured in the supernatant; formed lipid mediators were extracted by SPE and analyzed by UPLC-MS-MS. Amount of COX- or 5-LOX-derived lipid mediators and other monohydroxylated fatty acids (FAs) as well as polyunsaturated fatty acids (PUFAs) for unstimulated control and cells treated with vehicle, or 6BIGOE at 0.3 µM is shown in pg per 10^6^ cells. Data are absolute values represented as means ± SEM. Relative changes in the formation of single lipid mediators from monocytes treated with 6BIGOE versus vehicle-treated cells are displayed in a heat map. Color scheme indicates fold-change of mean values compared to mean of vehicle-treated cells (F_c_ = 1), n = 5 separate donors, except 6BIGOE: n = 4.

**Suppl. Figure S6. Influence of NP blanks on lipid mediator formation in human monocytes.** Human monocytes (10^6^/mL) were pretreated with vehicle (0.1% PBS (v/v)), or amount of blank NP dispersions prepared by emulsion-diffusion-evaporation (EDE), Cyrene, or PEG 400 method, equivalent to 6BIGOE-loaded NPs at 0.3 µM for 3 h before stimulation with 100 ng/mL LPS for 18 h at 37 °C. Lipid mediator formation was measured in the supernatant; formed lipid mediators were extracted by SPE and analyzed by UPLC-MS-MS. Amount of LM products for cells treated with vehicle or NP blanks at 0.3 µM is shown in pg per 10^6^ cells. Data are absolute values represented as means ± SEM. Relative changes in the formation of single lipid mediators from monocytes treated with NP blanks versus vehicle-treated cells are displayed in a heat map. Color scheme indicates fold-change of mean values compared to mean of vehicle-treated cells (F_c_ = 1), n = 5 separate donors.

**Suppl. Figure S7. Influence of 6BIGOE‑loaded NPs on lipid mediator formation in human monocytes.** Human monocytes (10^6^/mL) were pretreated with vehicle (NP blanks, shown in **Suppl. Fig. S5**), or 6BIGOE-loaded NPs prepared by emulsion-diffusion-evaporation (EDE), Cyrene, or PEG 400 method at 0.3 µM for 3 h before stimulation with 100 ng/mL LPS for 18 h at 37 °C. Lipid mediator formation was measured in the supernatant; formed lipid mediators were extracted by SPE and analyzed by UPLC-MS-MS. Amount of LM products for cells treated with 6BIGOE-loaded NPs is shown in pg per 1 x 10^6^ cells. Data are absolute values represented as means ± SEM. Relative changes in the formation of single lipid mediators from monocytes treated with 6BIGOE-loaded NPs versus NP blank-treated cells are displayed in a heat map. Color scheme indicates fold‑change of mean values compared to mean of NP blank-treated cells (F_c_ = 1), n = 5 separate donors.

**Suppl. Figure S8. Effects of NP blanks on COX‑2 expression in human monocytes.** Human monocytes (10^6^/mL) were pretreated with vehicle (0.1% PBS (v/v)), or blank NP dispersions prepared by emulsion-diffusion-evaporation (EDE), Cyrene, or PEG 400 method equivalent to 6BIGOE-loaded NPs at indicated concentrations for 3 h before stimulation with 100 ng/mL LPS for 18 h at 37 °C. COX-2 protein expression was assessed by Western Blot and densitometric analysis thereof, normalized to β-actin. Results are given as individual values represented in a box and whisker plot, n = 4 separate donors. Statistical analysis displayed was performed using one-way ANOVA for multiple comparisons with Holm‑Šídák post-hoc test.


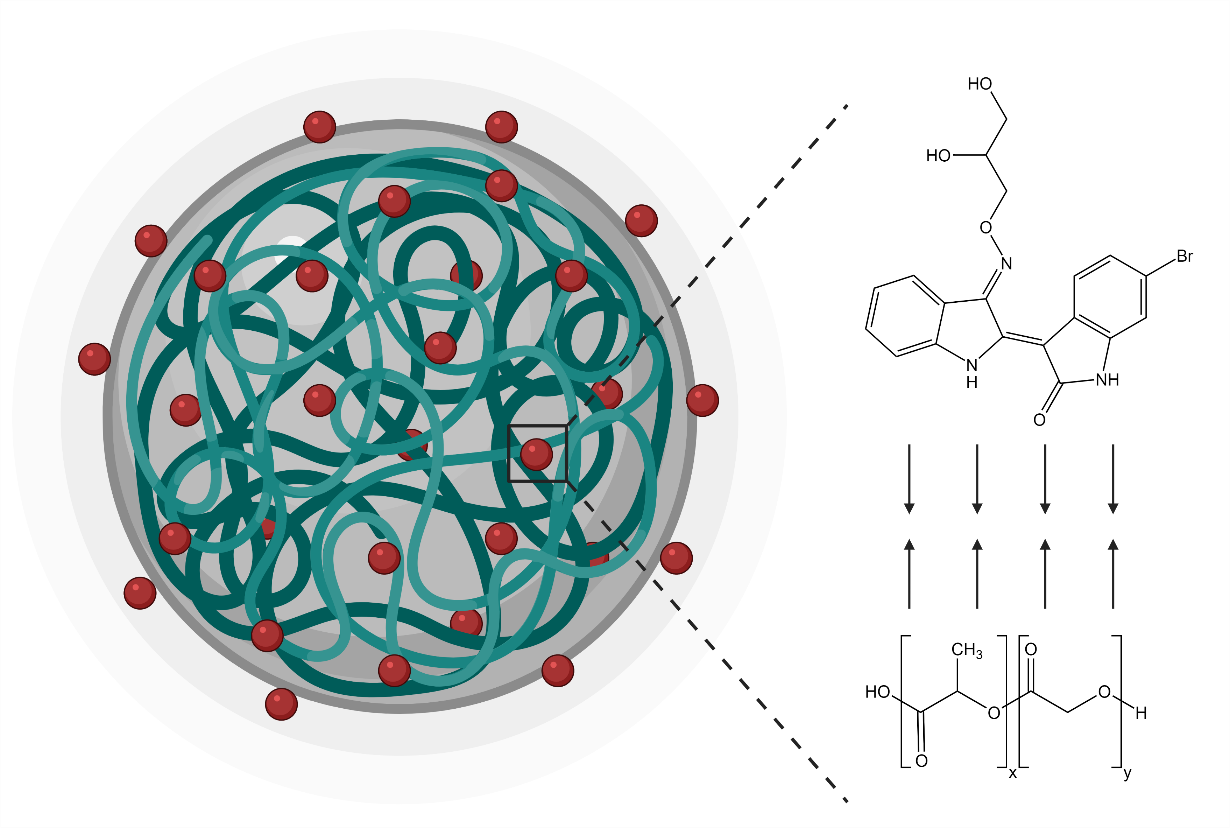


**Suppl. Figure S9.** **Interaction of 6BIGOE and PLGA.** Schematic representation of presumed localization of 6BIGOE (red) encapsulated within the PLGA matrix (blue) due to strong hydrophobic interactions.

**Suppl. Table S1.** Hydrodynamic diameter (HD), polydispersity index (PDI) and zeta potential (ZP) of FITC-PLGA-NPs loaded with and without 6BIGOE, prepared by emulsion-diffusion-evaporation method (EDE), PEG 400, or Cyrene method. Samples were analyzed by dynamic light scattering techniques in water (HD, PDI, ZP). Data were obtained from one batch.

| Preparation method | Sample | HD [nm] | PDI | ZP [mV] |
| --- | --- | --- | --- | --- |
| EDE | Blank NP | 198 | 0.0702 | -26.8 |
|  | 6BIGOE NP | 210 | 0.0912 | -19.1 |
| PEG 400 | Blank NP | 167 | 0.0886 | -24.3 |
|  | 6BIGOE NP | 197 | 0.197 | -19 |
| Cyrene | Blank NP | 218 | 0.0156 | -24.7 |
|  | 6BIGOE NP | 214 | 0.1288 | -20.2 |

**Suppl. Table S2.** Glass transition temperature (T_g_, °C) of PLGA, PLGA-blank-NP and 6BIGOE-loaded PLGA-NP prepared by EDE, PEG 400 or Cyrene method determined by differential scanning calorimetry in two heating-cooling cycles at 10 °C/min. T_g_ was calculated from run 2.

| Preparation method | Sample | T_g_ [°C] |
| --- | --- | --- |
|  | PLGA | 41.4 |
| EDE | Blank NP | 42.5 |
|  | 6BIGOE NP | 42.3 |
| PEG 400 | Blank NP | 33.9 |
|  | 6BIGOE NP | 42.3 |
| Cyrene | Blank NP | 41.2 |
|  | 6BIGOE NP | 42.1 |

**Suppl. Table S3.** IC_50_ values for modulation of pro-inflammatory cytokines (IL-1β, TNF‑α, IL-6) by 6BIGOE and 6BIGOE-NPs in LPS-stimulated human monocytes.

| cytokine | IC_50_ [µM] |  |  |  |
| --- | --- | --- | --- | --- |
|  | 6BIGOE | EDE | PEG 400 | Cyrene |
| IL-1β | 0.0349 | 0.0052 | 0.0061 | 0.0058 |
| TNF‑α | 0.0712 | 0.0337 | 0.0273 | 0.0408 |
| IL-6 | 0.0035 | 0.0047 | 0.0054 | 0.0061 |
